# Palaeobotanical evidence reveals the living conditions of Miocene *Lufengpithecus* in East Asia

**DOI:** 10.1186/s12870-023-04165-3

**Published:** 2023-03-22

**Authors:** Li-Li Lu, Yi-Feng Yao, Guo-An Wang, Gan Xie, Kai-Qing Lu, Bin Sun, Jin-Feng Li, Angela A. Bruch, David K. Ferguson, Yi-Ming Cui, Qiang Wang, Xin-Ying Zhou, Feng Gao, Yu-Fei Wang

**Affiliations:** 1 State Key Laboratory of Systematic and Evolutionary Botany, Institute of Botany, Chinese Academy of Sciences, 20 Nanxincun Xiangshan, 100093 Beijing, China; 2 University of Chinese Academy of Sciences, 100049 Beijing, China; 3 Department of Environmental Sciences and Engineering, College of Resources and Environmental Sciences, China Agricultural University, 100193 Beijing, China; 4 ROCEEH Research Centre, Senckenberg Research Institute and Natural History Museum, Senckenberganlage 25, 60325 Frankfurt, Germany; 5 Department of Paleontology, University of Vienna, Althanstrasse 14, A-1090 Vienna, Austria; 6 Lushan Botanical Garden, Chinese Academy of Sciences, 332900 Jiujiang, China; 7 Key Laboratory of Vertebrate Evolution and Human Origins, Institute of Vertebrate Paleontology and Paleoanthropology, Chinese Academy of Sciences, 100044 Beijing, China; 8 Department of Paleoanthropolpgy, Yunnan Institute of Cultural Relics and Archaeology, 650118 Kunming, Yunnan China

**Keywords:** Hominoid, *Lufengpithecus*, Habitat, Food web, Evolutionary fate

## Abstract

**Background:**

Understanding the relationship between human evolution and environmental changes is the key to lifting the veil on human origin. The hypothesis that environmental changes triggered the divergence of humans from apes (ca. 9.3–6.5 million years ago, Ma) has been poorly tested because of limited continuous environmental data from fossil localities. *Lufengpithecus* (12.5-6.0 Ma) found on the southeastern margin of the Tibetan Plateau (SEMTP) across the ape–human split provides a good chance for testing this hypothesis.

**Results:**

Here, we reconstructed the habitats of *L*. *keiyuanensis* (12.5–11.6 Ma) with comprehensive vegetation, climate, and potential food web data by palaeobotanical evidence, together with other multidisciplinary data and partly tested the environment-driven hypothesis by revealing the living conditions of *Lufengpithecus*.

**Conclusion:**

A detailed comparison of hominoids on different continents reveals their behaviour and fate divergence across the ape–human split against the background of global climate change, i.e., the stable living conditions of SEMTP not only provided a so-called ‘refuge’ for arboreal *Lufengpithecus* but also acted as a ‘double-edged sword’, preventing their further evolution while vegetation shifts in East Africa probably stimulated the emergence of human bipedalism, and the intense climatic changes in Europe possibly prevented those hominoids from surviving that time interval. Our findings provide interesting insight into the environmental impacts on the behavioural evolution of hominoids.

**Supplementary Information:**

The online version contains supplementary material available at 10.1186/s12870-023-04165-3.

## Background

The time, place and driving mechanisms of the ape–human split have been explored as topical issues for more than a century since Huxley’s initial assumption that humans and apes diverged from a common ancestor [1] and Darwin’s inference that the last common ancestor of *Pan* and *Homo* originated in Africa [2]. The human–chimpanzee split was inferred to have occurred in Africa ca. 9.3–6.5 million years ago (Ma) [3] by integrating the oldest hominin fossil [4] and genomic evidence (Additional file 2: Fig. S1) [5, 6]. With regard to the mechanisms, several hypotheses have emphasized environmental changes as an evolutionary driver of this significant divergence process. For instance, the habitat-specific hypothesis claims that the savanna was the context stimulating the emergence of terrestrial bipedality, larger brains, stone tool-making, meat-eating, and associated foraging behaviours such as hunting [7–9]. The variability selection hypothesis, which could be explained by early hominin bipedality, states that certain adaptations have evolved under intense environmental selection pressures [10–12]. However, the largest problem in testing these hypotheses with data concerns the poor connection between hominoid fossils and their corresponding palaeoenvironments [11, 13].

*Lufengpithecus* (12.5-6.0 Ma) [14, 15], a primitive hominid sister to Ponginae and Homininae [16, 17] or a primitive sister taxon of Ponginae [18, 19] found on the southeastern margin of the Tibetan Plateau (SEMTP) and dated to the period of the ape–human split (~ 9.3–6.5 Ma), might represent a resource for testing the abovementioned hypotheses.

By integrating multidisciplinary evidence from palaeobotany, geochemistry, palaeomammalogy, and sedimentology, we reconstructed the palaeovegetation, palaeoclimate, and potential food web during the period of *Lufengpithecus keiyuanensis* occurrence (the earliest species of *Lufengpithecus*, 12.5–11.6 Ma) [14, 20] and further revealed the living conditions of Miocene *Lufengpithecus* in East Asia.

**Fig. 1:**
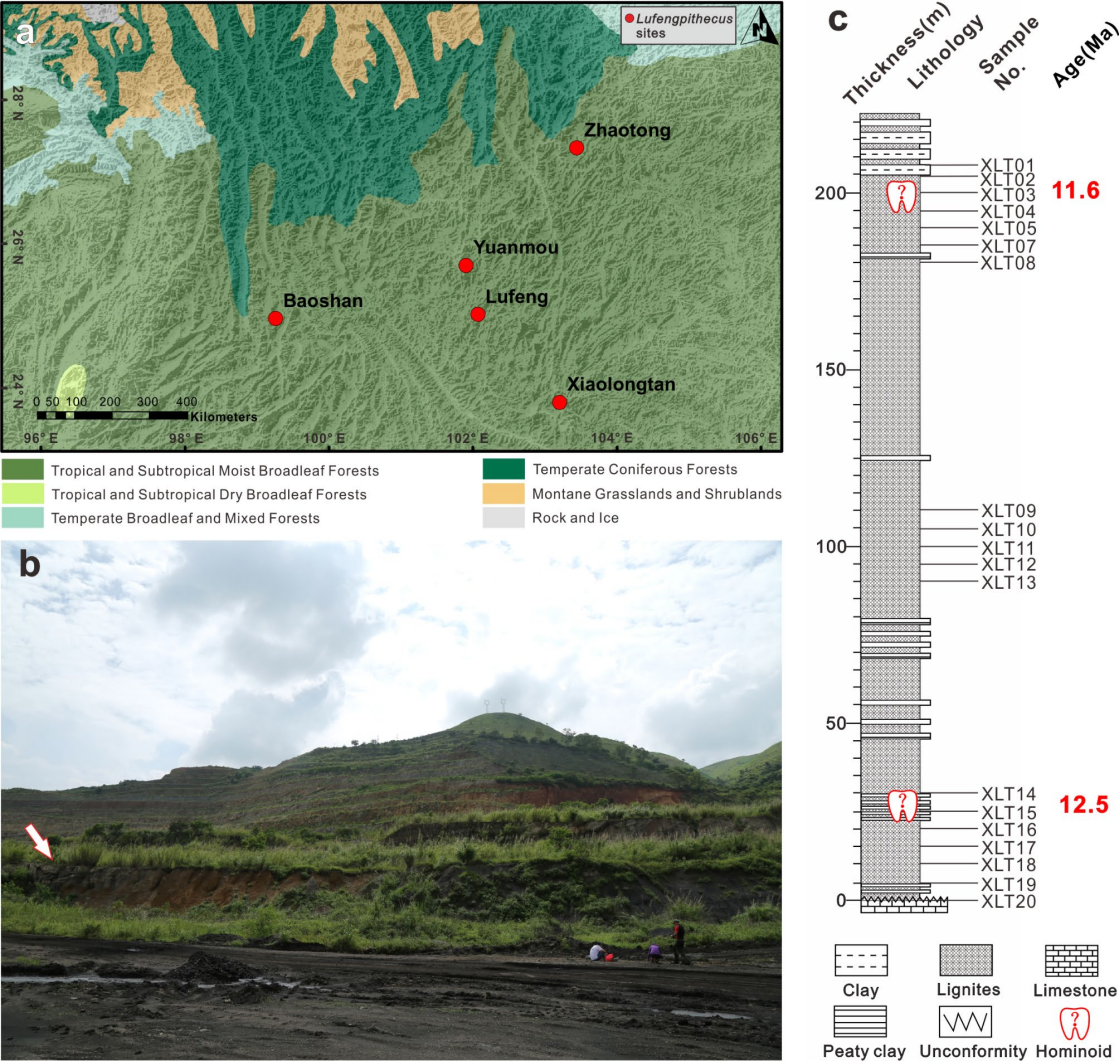
Miocene* Lufengpithecus* distribution and geological data for the sampling profile of* Lufengpithecus keiyuanensis*. (**a**) The study area showing the geographical locations of *Lufengpithecus* fossil sites (red spots) on the SEMTP on a map of terrestrial biome ecoregions [21]. (**b**) Photos of the sampling section (below the arrow) in the Xiaolongtan coal mine. (**c**) The measured stratigraphic sequence. (modified from ref.14) and pollen sampling sequence of the Xiaolongtan section

## Results

### Palaeobotanical data

**Fig. 2:**
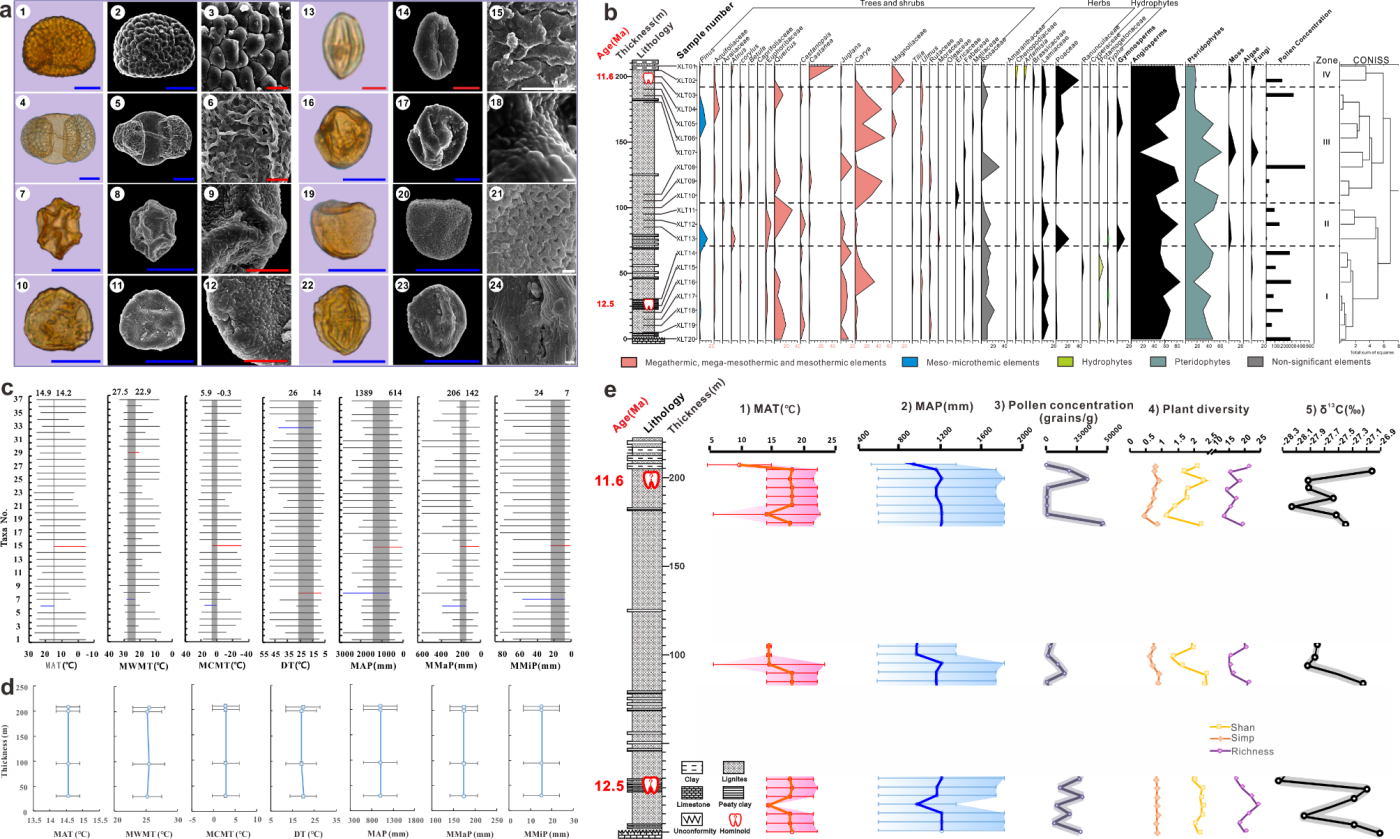
The vegetation and climate during the *L. keiyuanensis * era. (**a**) Photomicrographs of selected single pollen grains. Blue scale = 20 μm; red scale = 5 μm; white scale = 1 μm. 1–3. Polypodiaceae; 4–6. *Pinus*; 7–9. *Alnus*; 10–12. *Carya*; 13–15. *Castanopsis*; 16–18. Fabaceae; 19–21. *Typha*; 22–24. Anacardiaceae. (**b**) Diagram showing changes in the relative abundances (expressed as %) of the major palynomorphs recovered from the Xiaolongtan section. (**c**) Coexisting intervals of palynological assemblages from the Xiaolongtan section. The upper boundary of climatic parameters is the red line segment, and the lower boundary is the blue line segment. (1) *Alnus*; (2) Aquifoliaceae; (3) Araliaceae; (4) *Artemisia*; (5) Asteraceae; (6) *Betula*; (7) *Carya*; (8) *Castanea*; (9) *Castanopsis*; (10) Chenopodiaceae; 11. *Corylus*; 12. Brassicaceae; 13. Cyperaceae; 14. Elaeagnaceae; 15. *Ephedra*; 16. Ericaceae; 17. Euphorbiaceae; 18. Fabaceae; 19. *Juglans*; 20. Lamiaceae; 21. Magnoliaceae; 22. Meliaceae; 23. Moraceae; 24. Oleaceae; 25. *Pinus*; 26. Poaceae; 27. Potamogetonaceae; 28. *Quercus*; 29. Ranunculaceae; 30. Rosaceae; 31. Rutaceae; 32. Sapindaceae; 33. Solanaceae; 34. Taxodiaceae; 35. *Tilia*; 36. *Typha*; 37. *Ulmus.* (**d**) Climatic parameters of individual horizons (Zones I-IV) from the Xiaolongtan section estimated by CoA. (**e**) The measured stratigraphic sequence of the Xiaolongtan Sect. [14] and the palaeotemperature, palaeoprecipitation, pollen concentration, plant diversity, and stable carbon isotopes. (1) MAT; (2) MAP; (3) Pollen concentration; (4) Plant diversity; (5) δ^13^C

The Miocene Xiaolongtan (XLT) section (23°48.351′N; 103°10.410′E, 1105 m above sea level (a.s.l.), Fig. 1a-b) bearing the fossil of *L. keiyuanensis* contains a highly diverse palynomorph assemblage of forty-three pollen and spore taxa (see more details in Fig. 2a, Additional file 3: Fig. S2, Additional file 4: Fig. S3, Additional file 5: Fig. S4 and Additional file 8: Table S1). The assemblage is dominated by pollen of woody plants (49.6%, mainly *Carya*, Rosaceae, *Quercus*, *Juglans*, *Castanopsis*, and *Castanea*) and herbs/grasses (12.6%, mainly Poaceae and Lamiaceae) with abundant spores of ferns (32.9%), including Polypodiaceae and Athyriaceae during the existence of *L. keiyuanensis*. The total pollen/spore concentration varied little during this period, averaging more than 10,000 grains/gram (Fig. 2e), indicating a period of abundant plant diversity and dense forests.

According to the temperature preferences of the nearest living relatives [22], the Xiaolongtan palynomorphs included 5 megathermic elements (Aquifoliaceae, Meliaceae, Rutaceae, Sapindaceae, and Solanaceae), 4 mega-mesothermic elements (Araliaceae, *Castanopsis*, Euphorbiaceae, and Taxodiaceae), 13 mesothermic elements (e.g., *Betula, Carya, Castanea, Corylus, Juglans, Quercus*), one meso-microthermic element (*Pinus*), 1 climatically non-significant element (Rosaceae), and 10 herbs and/or shrubs (e.g. *Artemisia*, Chenopodiaceae, and *Ephedra*) (Additional file 9: Table S2).

Furthermore, except for evergreen broad-leaved pollen identified under the light microscope such as *Castanopsis*, typical evergreen oak pollen with rod-like ornamentation could be identified based on the observation of two grains of *Quercus* pollen under the scanning electron microscope (Additional file 5: Fig. S4) [23]. Therefore, it could be inferred that evergreen broad-leaved trees existed in this vegetation.

### Palynological diagram

Based on the changes in the major palynomorphs and their relative abundance from the Xiaolongtan section, the pollen diagram (Fig. 2b) could be divided into four zones by constrained incremental sums of squares (CONISS) cluster analysis conducted in TILIA. The changes in each zone are as follows:

Zone I (0–30 m, XLT20-XLT14). This zone contained 30 palynomorphs of angiosperms (49.9%), 2 palynomorphs of gymnosperms, 5 palynomorphs of pteridophytes, and a small number of mosses, algae, and fungal spores.

Rosaceae as a widely distributed component had the highest pollen abundance of 13.1% in angiosperms. *Quercus* (9.6%), *Juglans* (9.1%), and *Carya* (8.9%) were dominant in woody angiosperms (49.9%). Gymnosperms included a small amount of *Pinus* (0.57%) and Taxodiaceae (0.1%), which prefer relatively warm and wet environments. The average abundance of ferns was as high as 35.4%, and Polypodiaceae (27.4%) was dominant.

In this zone, there were 5 megathermic elements (Aquifoliaceae, Meliaceae, Rutaceae, Sapindaceae, and Solanaceae); 4 mega-mesothermic elements (Araliaceae, *Castanopsis*, Euphorbiaceae, and Taxodiaceae); 10 mesothermic elements (e.g. *Betula*, *Carya*, *Castanea, Corylus, Juglans, Quercus*); 1 meso-microthermic element (*Pinus*); 1 climatically non-significant element (Rosaceae); 10 herbs and/or shrubs; and 2 aquatic macrophytes (Potamogetonaceae and *Typha*). The pollen concentration (18,605 grains/g on average) was the highest in this zone, which may indicate that the vegetation coverage was very dense during this period.

Zone II (85–95 m, XLT13-XLT11). This zone contained 26 palynomorphs of angiosperms, 1 palynomorph of gymnosperms, 4 palynomorphs of pteridophytes, and a small number of mosses, algae, and fungal spores.

Compared with Zone I, the relative abundance of woody angiosperms decreased slightly (45.5%). The amount of *Quercus* (16.4%) increased sharply and became the dominant component, while Rosaceae (8.8%), *Carya* (1.7%), and *Juglans* (3.8%) decreased. Concurrently, herbaceous taxa such as Poaceae (7.4%) and Lamiaceae (6%) increased. The only gymnosperm was *Pinus* (4.4%). The abundance of pteridophytes decreased slightly (30.5%), while Polypodiaceae (23.3%) was still predominant. The pollen concentration (8,365 grains/g on average) fell to a minimum, which might indicate that the vegetation coverage decreased in this period.

In this zone, there were 4 megathermic elements (Aquifoliaceae, Meliaceae, Rutaceae, and Sapindaceae); 3 mega-mesothermic elements (Araliaceae, *Castanopsis*, and Euphorbiaceae); 9 mesothermic elements (e.g., *Alnus, Betula, Carya, Castanea, Juglans, Quercus*); 1 meso-microthermic element (*Pinus*); 1 climatically non-significant element (Rosaceae); 10 herbs and/or shrubs; and 2 aquatic macrophytes (Potamogetonaceae and *Typha*).

Zone III (100–200 m, XLT10-XLT03). This zone contained 29 palynomorphs of angiosperms, 2 palynomorphs of gymnosperms, palynomorphs of pteridophytes, and a small number of mosses, algae, and fungal spores.

Compared with Zone II, the relative abundance of woody angiosperm pollen increased to 51%. *Carya* (26.3%) became the dominant tree group in the vegetation, while *Quercus* (3.6%) and *Juglans* (3.1%) decreased. Rosaceae decreased further to 6.3%. Herbaceous angiosperms decreased to 7.9%, such as Poaceae (4.8%) and Lamiaceae (2.3%). In gymnosperms, *Pinus* (2.4%) decreased slightly, while *Ephedra* (0.1%) appeared for the first time in this zone with low relative abundance. Pteridophyte (33.4%) spores increased, and Polypodiaceae (30.5%) remained predominant. The pollen concentration (10,995 grains/g on average) increased again.

In this zone, there were 3 megathermic elements (Aquifoliaceae, Meliaceae, and Rutaceae); 2 mega-mesothermic elements (*Castanopsis* and Euphorbiaceae); 11 mesothermic elements (e.g., *Alnus, Betula, Carya, Castanea, Juglans, Quercus*); 1 meso-microthermic element (*Pinus*); 1 climatically non-significant element (Rosaceae); 10 herbs and/or shrubs; and 2 aquatic macrophytes (Potamogetonaceae and *Typha*).

Zone IV (205–208 m, XLT02-XLT01). There were 18 palynomorphs in angiosperms, 3 palynomorphs in gymnosperms, 3 palynomorphs in pteridophytes, and spores of bryophytes, algae, and fungi.

Woody angiosperm (44.3%) pollen decreased slightly. *Castanea* (20.6%) became the dominant taxon in this zone, followed by Magnoliaceae (12%), while *Carya* (0.8%), *Quercus* (0.5%), and *Juglans* (1.1%) decreased to varying degrees, with Betulaceae pollen disappearing altogether. Herbaceous angiosperms increased significantly (33%), including Poaceae (22.5%) and Lamiaceae (4.9%). Gymnosperms were still not high, with *Pinus* decreasing slightly (0.6%), *Ephedra* increasing slightly (0.2%), and Taxodiaceae accounting for 0.2%. Pteridophyte spores fell to 22.8%, and the amount of Polypodiaceae also decreased (15.1%). The pollen concentration (9,429 grains/g on average) decreased slightly in this zone.

There were 2 megathermic elements (Aquifoliaceae and Solanaceae); 1 mega-mesothermic element (Taxodiaceae); 8 mesothermic elements (e.g., *Alnus, Carya, Castanea, Juglans, Quercus*); 1 meso-microthermic element (*Pinus*); 1 climatically non-significant element (Rosaceae); 7 herbs and/or shrubs; and 1 aquatic macrophyte (*Typha*).

### Palaeovegetation

The pollen assemblages reflect that the vegetation during the period of occurrence of *L. keiyuanensis* was a subtropical deciduous and evergreen broad-leaved mixed forest represented by the families Fagaceae and Juglandaceae.

**Fig. 3:**
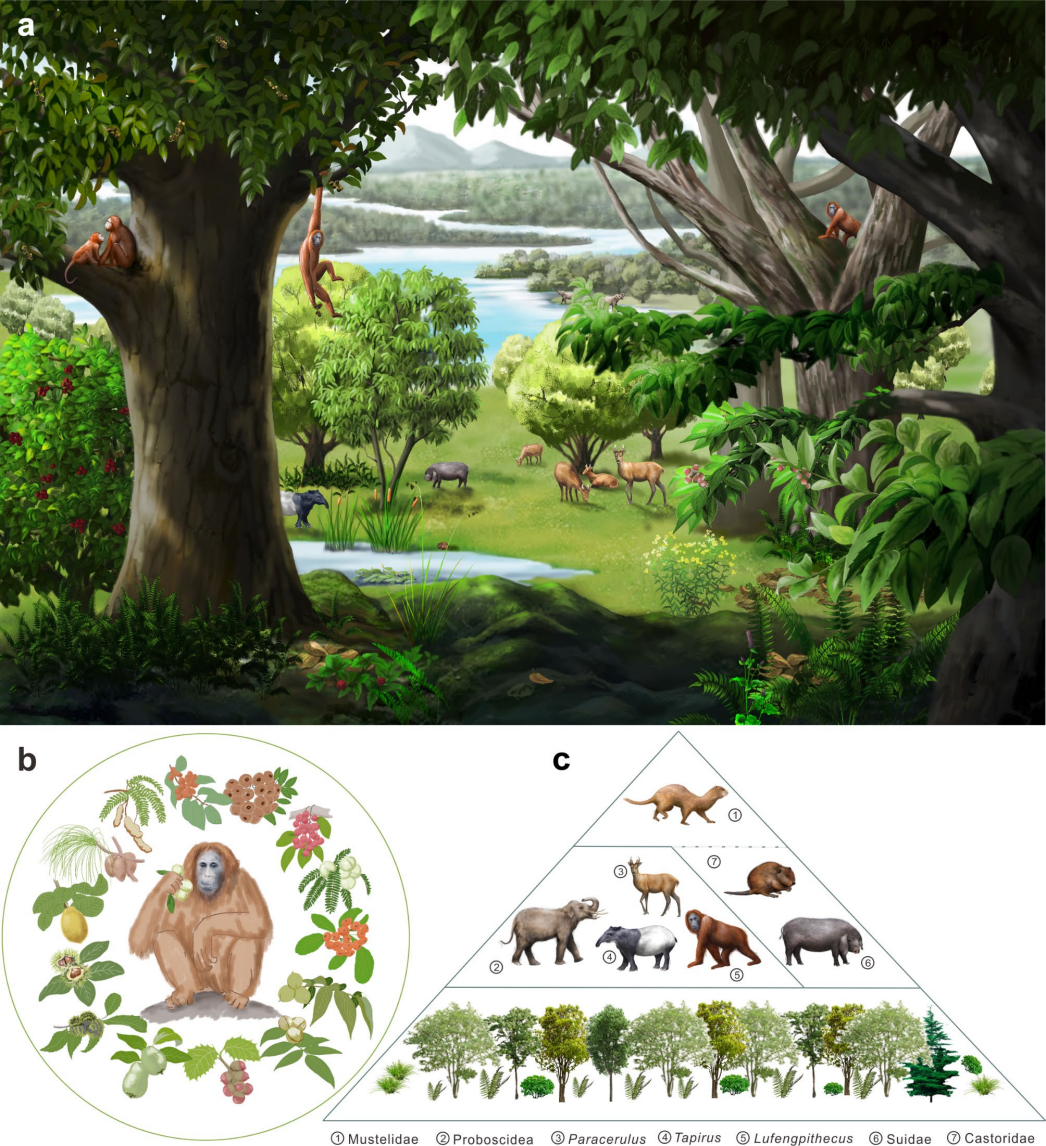
The living conditions of* L. keiyuanensis* showing vegetation, climate, and potential food resources. (**a**) Reconstruction of the living environment and ecosystem of *L. keiyuanensis.* The painting is based on palaeofloral, palaeomammalian, and environmental information inferred from palynological, geochemical, sedimentological, and mammalian fossil evidence obtained from the Middle Miocene Xiaolongtan Basin. (**b**) Diet composition. The painting is based on the composition of major fodder plants inferred from the palynomorph assemblage and mega-fossils [27] from the Xiaolongtan Formation. These are arranged clockwise in order of the season of fruit and seed season maturity today [28]. (**c**) Energy pyramid

Although four stages of vegetation succession could be distinguished over this time interval (see more details in Additional file 1: Additional information and Additional file 6: Fig. S5), the vegetation appears to have been generally stable (Fig. 3a), and the changes at each stage were mainly confined to community composition and the relative abundance of dominant taxa (Zone I: *Quercus* (9.6%), *Juglans* (9.3%), and *Carya* (9%); Zone II: *Quercus* (12.8%); Zone III: *Carya* (27.3%); Zone IV: *Castanea* (21.1%) and Magnoliaceae (12.2%)).

Based on previous studies [14, 24, 25], the excavation position of the *L. keiyuanensis* fossils may be located at the upper or lower part of the lignite, roughly corresponding to either Zone I or III. Therefore, here, we provide a further description of the palaeovegetation of these two zones. In Phase 1 (corresponding to Zone I), the evergreen and deciduous broad-leaved mixed forests were largely composed of woody plants such as *Quercus*, *Juglans*, and *Carya*, which are common forests taxa in temperate to subtropical areas today. In Phase 3 (corresponding to Zone III), the forest was dominated by *Carya* with small numbers of *Quercus, Juglans, Castanea*, and *Ulmus*, as well as occasional *Castanopsis* and *Alnus*.

In a word, both palynological assemblages of Zones I and III indicate evergreen and deciduous broad-leaved mixed forests while their differences are depicted as that *Quercus* (9.6%), *Juglans* (9.1%) and *Carya* (8.9%) were dominant in Zones I and *Carya* (26.3%), *Quercus* (3.6%) and *Juglans* (3.1%) were the dominant tree group in Zones III (see more detailed data in Additional file 10: Table S3).

### Palaeoclimate

Based on 37 fossil pollen taxa, 7 climatic parameters were reconstructed by the coexistence approach (CoA) for the whole pollen assemblage and each pollen zone (see more details in Additional file 1: Additional information and Additional file 11: Table S4, Fig. 2c-e). The climatic estimations show stable conditions for all assemblages, with a mean annual temperature (MAT) of 14.2–14.9 °C (median 14.6 °C) and a mean annual precipitation (MAP) of 614–1389 mm (median 1000 mm), indicating that the Middle Miocene conditions were significantly cooler and wetter than present conditions (MAT: 20.1 °C, MAP: 770 mm) and were favourable for maintaining relatively high and stable floristic diversity throughout the 12.5 to 11.6 Ma period (Figs. 2e and 3a).

### Stable carbon isotopes

Carbon isotope analysis is one of the important indices for the reconstruction of the palaeoenvironment [26]. The δ^13^C record from the Xiaolongtan section (see more details in Additional file 1: Additional information, Fig. 2e) corresponds well to the vegetation and climate signals, suggesting that the local vegetation was dominated by C_3_ plants and that the MAP was at least 1000 mm. Moreover, the variations in δ^13^C values throughout the section were not very large (-27.621 ± 1.4‰), which indicates that the climate was relatively stable with small fluctuations.

## Discussion

Compiling palaeobotanical data together with other multidisciplinary evidence from geochemistry, palaeomammalogy, and sedimentology and a set of continuous climatic data provided here, we interpreted the living conditions of *Lufengpithecus* by addressing three questions as follows.

### Where to live?

*L. keiyuanensis* lived in a warm and moist subtropical evergreen and deciduous broad-leaved mixed forest in montane valleys [24], where the dominant trees would have been *Castanopsis*, *Quercus*, and *Carya.* Thermophilous plants such as Aquifoliaceae, Elaeagnaceae, and Euphorbiaceae grew interspersed with lush bushes. Ferns flourished in the shade. Aquatic plants such as *Typha*, Potamogetonaceae, and Cyperaceae occur in the palaeolake and surrounding wetlands. Xerophilic *Ephedra* and Chenopodiaceae could be found in exposed areas away from jungle and water. *Pinus* stood on higher ground in the distance. Abundant mammals lived in this type of forest from the Middle to Late Miocene, including *L. keiyuanensis*, Mustelidae indet., Castoridae gen. et sp. indet., *Tetralophodon xiaolongtanensis*, *Gomphotherum* cf. *macrognathus*, *Zygolophodon chinjiensis*, *Tapirus* cf. *yunnanensis*, *Parachleuastochoerus sinensis*, *Propotamochoerus parvulus*, *Hippopotamodon hyotherioides* and *Euprox* sp. [25]. The analogues of those ecological habits of the nearest living mammal relatives might suggest that large mammals such as proboscideans stroll through the forest feeding on plants, tapirs may push their way through the undergrowth, omnivorous pigs could root in search of food, deer may move nimbly through dense jungle, apes probably gather fruit from tree canopies, beavers can dam streams, and tiny carnivorous weasels could be quick-witted in search of prey (Fig. 3a).

The palaeoclimate data of the top layer of the Xiaolongtan Formation bearing plant megafossils was reported by Xia et al., 2009 [27]. Here our work provided the palaeoclimate data of the *L. keiyuanensis* fossil layers of the Xiaolongtan Formation. The detailed quantitative climatic data of both works were somewhat different, which may come from the different methods used by both Xia et al. and our study to reconstruct the palaeoclimate based on various delimited plant taxa; however, results of both studies indicated a warm and humid subtropical climate (see more details in Additional file 12: Table S5).

### What to eat?

Food is the link between hominoids and their environments. Food sources determine the feeding behaviour and the course of evolution in hominoids [29]. Feeding adaptations in primates also reflect key behavioural and ecological differences to some extent [29, 30]. All great apes are largely plant-eating and consume varying proportions of fruits and leaves [29].

The cranium and postcranium of *Lufengpithecus* point to an arboreal habit [31, 32]. The correlation between the development of cutting ridges on the occlusal surface of molars and the feeding habits of living primates reveals that primates with poor cutting ridges mostly feed on hard fruits, while primates with well-developed cutting ridges mainly feed on soft foods such as leaves and buds [33]. Apparently, *Lufengpithecus* mainly fed on fruit [34] and was adapted to eating hard foods that require chewing and grinding, according to its underdeveloped molar cutting ridges and relatively thick enamel layer [35, 36].

According to the optimal foraging theory, i.e., obtaining the maximum energy intake in the shortest possible time [37], *Lufengpithecus* likely preferred to exploit the dominant species of forests within its habitat range. In the forest, there were lipid-rich drupes from *Juglans* and *Carya*, starchy nuts from *Quercus, Castanea*, and *Castanopsis*, and vitamin-rich and cellulosic fruits of Rosaceae (Fig. 3b). Indeed, the potential vital food sources of *Lufengpithecus* might have been nuts and drupes, based on the dominant taxa in the palaeobotanical record from three other fossil sites (Additional file 13: Table S6).

By considering the feeding habits of nonhuman primates today, it is possible to speculate on the potential seasonal food sources of *L. keiyuanensis* (Fig. 3b). In spring, tender leaves and buds had higher protein and water contents, so *L. keiyuanensis* may have eaten mainly young leaves. In summer, the number of such food sources decreased, forcing *L. keiyuanensis* to feed more on mature leaves and the first ripe fruits of Rosaceae available at this time. Autumn was a period of plenty, with drupes and nuts becoming the main foods of *L. keiyuanensis.* In winter, deciduous plants stopped growing, so the number of edible plant parts may have decreased; however, many nuts and drupes as well as fruits of Rosaceae (e.g., rose hips) remained on the plant in winter and must have been available for exploitation by *L. keiyuanensis*.

Were there any natural enemies in the habitats of *L. keiyuanensis*? The skull of a 4-Ma *Australopithecus*, which is a distant relative of *Lufengpithecus*, showed tooth marks from a leopard [9]. Fossil evidence from Yuanmou suggests that large cats might have been regular predators of *L. hudienensis* [38]. In Xiaolongtan, the only carnivore found thus far was a kind of small, widespread weasel associated with *L. keiyuanensis*. Admittedly, due to the limitations of fossil preservation and distribution, the presence of other large carnivores in the Xiaolongtan ecosystem cannot be ruled out [24]. However, according to the evidence, *L. keiyuanensis* would have had few natural enemies.

The energy pyramid (Fig. 3c) of *L. keiyuanensis* could probably be divided into three trophic levels: the primary producers were members of subtropical evergreen and deciduous broad-leaved forests, which supplied the fundamental source of energy for the survival of all mammals (Fig. 3a), with *Carya, Quercus*, and *Juglans* as the dominant taxa; primary consumers occupied the middle level, which included *L*. *keiyuanensis*, herbivores (three species of elephants, one tapir, one deer) and omnivorous animals (three species of pigs and one beaver); secondary consumers included omnivorous animals and the only carnivore, the weasel.

### Fate, where to go?

**Fig. 4:**
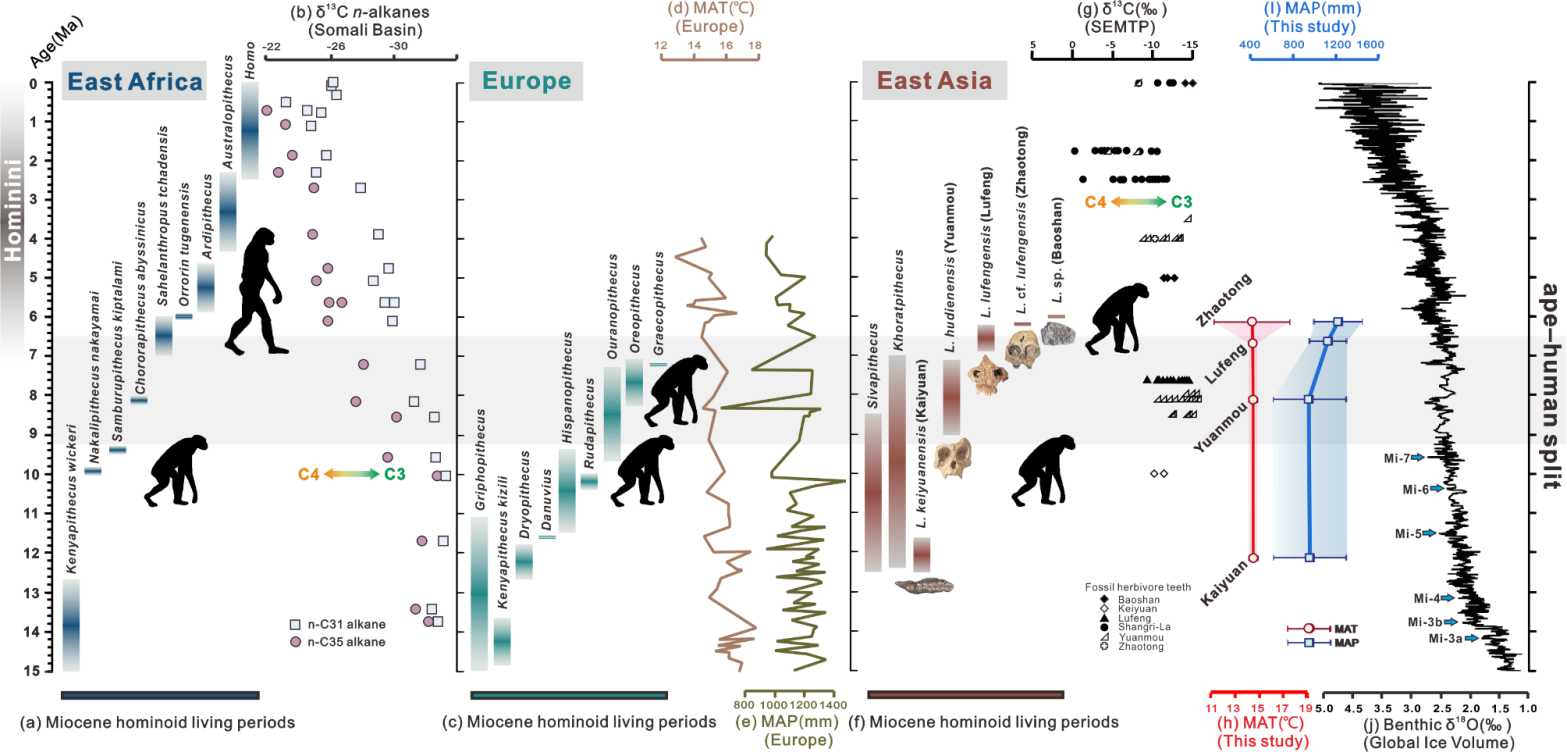
Occurrence dates, behavioural patterns and environments of hominoids in East Africa, Europe and East Asia since the Middle Miocene. (**a**) Occurrence dates of hominoids in East Africa [49]. (**b**) Carbon isotopic data from Somali Basin cores showed the onset of expansion of C_4_ vegetation at 10 Ma [49]. (**c**) Occurrence dates of hominoids in Europe [40]. (**d**) Integrated European MAT change curves [54]. (**e**) Integrated European MAP change curves [54]. (**f**) Occurrence dates of hominoids in East Asia [3, 9]. (**g**) Carbon isotopic changes at various locations in Yunnan show the onset of expansion of C_4_ vegetation at approximately 4 − 3 Ma [47]. (**h**) MAT reconstruction results for four *Lufengpithecus* sites. (**i**) MAP reconstruction results for four *Lufengpithecus* sites. (**j**) Deep sea oxygen isotopes indicative of global ice yield [43] and a series of Miocene cooling events [42].

The SEMTP, East Asia, was suggested as a ‘refuge’ [15, 18, 39] for the numerous species of *Lufengpithecus* in the Late Miocene (12.5-6.0 Ma), while most hominoids disappeared in Africa [9] and Europe [40] during that time against the background of marked global cooling and several climatic fluctuations (Fig. 4j) [41–44].

Palaeobotanical evidence from four sites of *Lufengpithecus* (Kaiyuan, Yuanmou, Lufeng [45], and Zhaotong [46]) indicates that arboreal *Lufengpithecus* lived in a warm, humid, and stable subtropical forest environment with few climatic fluctuations (median MAT ~ 14.5 (11.3–17.6) °C, median MAP ~ 1080 (614–1547) mm, see Fig. 4h-i, Additional file 14: Table S7 and Additional file 15: Table S8). This dense forest habitat was maintained until 4 − 3 Ma (Fig. 4g) [47]. A region with such a long period of stable environmental conditions will undoubtedly act as a ‘refuge’, while other environments will become unstable. However, this stability could have acted as a ‘double-edged sword’, depriving *Lufengpithecus* of external drivers and possibilities for subsequent adaptation and evolution during divergence.

In Africa, a vegetation shift from forest to savanna driven by aridification might have triggered the behavioural transition of hominoids. Evidence has shown that savanna/grassland expansion in East Africa occurred approximately 10 Ma (Fig. 4b) [48, 49], controlled by MAP [50]. In response to the subsequent selection pressures of a reduced distribution range, tightened food sources, and increased intra- and interspecific competition, primitive arboreal hominoids probably developed some key adaptive traits to cope with these changes, such as bipedality, toolmaking, meat-eating, and brain expansion [8], which resulted in the evolution of bipedal humans from arboreal apes, i.e., the human–chimpanzee split (~ 9.3–6.5 Ma, Fig. 4 and Additional file 2: Fig. S1). Fossil evidence suggests that *Sahelanthropus tchadensis* and *Orrorin tugenensis* subsequently emerged as possible and probable early hominins at approximately 7 − 6 Ma (Fig. 4a) [51].

In Europe, increased aridification (Fig. 4d-e) [52–54] and seasonality [52] during the Late Miocene and thereafter might have exceeded the survival threshold for arboreal hominoids during that divergence period [40], which might have been the main reason for their extirpation. Traces of hominoids from the Middle Miocene have been found, and profound adaptive radiation followed (Fig. 4c). After ca. 9.5 Ma, hominoids are no longer recorded from Western and Central Europe—except for *Oreopithecus*, which survived until ca. 7 Ma in its insular refuge (Fig. 4c) [40].

## Conclusion

We first extracted pollen data from *Lufengpithecus* fossil localities across the ape–human split by using standard methods for sampling, experiments, photography, and statistics. Then, we obtained climatic parameters by using the coexistence approach analysis on pollen data. Our results quantitatively revealed for the first time how the SEMTP could have been a ‘refuge’ for Miocene hominoids.

Here we partly tested the environment-driven hypothesis by revealing the living conditions of *Lufengpithecus* based on habitat reconstruction of *L*. *keiyuanensis* with comprehensive vegetation, climate, and potential food web data by palaeobotanical evidence, together with other multidisciplinary data. We found that the relatively stable subtropical climate of the SEMTP not only provided a ‘refuge’ for arboreal *Lufengpithecus* but also acted as a ‘double-edged sword’, obstructing its further evolution against the background of global cooling across the ape–human split based on the comparison of hominoids on different continents.

These findings provide an interesting case for partly testing the environment-driven hypothesis regarding the ape–human split. Nevertheless, we should note that our understanding of the environmental impacts on the divergence of humans from apes is still very limited. Additional quantitative climatic reconstructions for the ape existing period remain urgently needed in Africa and Europe. Increasing amounts of environmental data, as presented here for *Lufengpithecus*, from fossil sites that cover the period of the ape–human split will greatly contribute to our knowledge of hominoid morphological and behavioural evolution.

## Methods

### Sampling

*L. keiyuanensis* was excavated from the Xiaolongtan Basin which is near the Tropic of Cancer and has a south subtropical monsoon climate today. The local vegetation of this basin is subtropical monsoon evergreen broad-leaved forest with Fagaceae and Lauraceae as the dominant groups in the canopy [55] and with a MAT of 20.1 °C and a MAP of 770 mm (http://data.cma.cn/).

Thirteen teeth and a maxilla with 12 teeth of hominoid were recovered successively in the Xiaolongtan Basin from 1956 to 1982 [56–58]. There are two opinions regarding the excavation position of this hominoid fossil, the upper part of the lignite, 20–30 m below the upper mudstone [24, 25], dated ca. 11.6 Ma [14], or the lower part of the lignite, ~ 27 m in our section, dated ca. 12.5 Ma [14]. Twenty pollen samples were collected from the top to the bottom of the section, including the two known potential hominoid positions (Fig. 1b-c). These pollen samples have been deposited in the Palaeobotanical Museum of China, Institute of Botany, Chinese Academy of Sciences, under specimen numbers XLT 01-XLT 20.

The *L. hudienensis* skull was excavated in the Yuanmou (YM) Basin (Additional file 7: Fig. S6, 25°54.792′N, 101°46.124′E, 1142 m a.s.l.) [17] which belongs to the dry-hot valley climate type. Four pollen samples were collected from the skull-bearing horizon of this section (Additional file 7: Fig. S6). The palynological data in this section are provided in Additional file 1: Additional information, and the relative abundance of palynomorphs recovered from the section is shown in Additional file 16: Table S9. These pollen samples were deposited in the Palaeobotanical Museum of China, Institute of Botany, Chinese Academy of Sciences, under specimen numbers YM 01-YM 04.

### Palynological analysis

We used the heavy liquid separation method (density: 1.9 g/ml) [59, 60] to extract pollen and spores from the above samples. One tablet of *Lycopodium* spores (batch: 2,013,001; 27,560 ± 2643 spores/tablet) was added to each sample before treatment to calculate the pollen concentration. At least 300 spores and pollen grains were counted for each sample, in addition to *Lycopodium* spores, under a Leica DM 2500 light microscope at a magnification of 400×. The identification of the palynomorphs was identified by comparison with palynological monographs [61–63]. TILIA 1.7.16 software was used to calculate the relative abundance, pollen concentration, and pollen diagram plotting. The division of pollen zones was obtained by constrained incremental sums of squares (CONISS) cluster analysis using TILIA 1.7.16 (https://www.tiliait.com/).

Using the coexistence approach (CoA) [64] analysis of pollen data, we obtained the following climatic parameters for the Xiaolongtan Basin and Yuanmou Basin: the mean annual temperature (MAT), the mean warmest monthly temperature (MWMT), the mean coldest monthly temperature (MCMT), the temperature difference between the coldest and warmest months (DT), the mean annual precipitation (MAP) the mean maximum monthly precipitation (MMaP), and the mean minimum monthly precipitation (MMiP).

The palaeoclimatic parameters (MAT and MAP) in Lufeng were also quantitatively reconstructed by using the CoA of the pollen assemblages from the hominoid layer [45].

During the calculation of the numerical ranges of palaeoclimatic parameters using the CoA, we referred to the mean annual temperature variation ranges of the nearest recent relatives (NRLs) of fossil plants in the Palaeoflora Database (http://www.geologie.uni-bonn.de/Palaeoflora_home.htm) and collected the modern climatic data of the distribution regions of each taxon [65] based on the Surface Meteorological Data of China (1951–1980) [66]. Then, we superimposed the climatic data of all distribution areas of each taxon and calculated the range of climatic parameters. Finally, we calculated the coexistence intervals of the climatic parameters of all plant taxa (Fig. 2c-e, Additional file 11: Table S4 and Additional file 15: Table S8).

### Evaluating plant diversity

The abundant plant taxa in the forest supplied diverse food sources during the *L*. *keiyuanensis* era. The plant diversity of each sample was measured using palynological data (all palynological types identified in the samples, including ferns and aquatic plants) to investigate the changes in plant diversity and its response to climatic change during the living period of *L*. *keiyuanensis*. Plant diversity (Simpson index, Shannon‒Wiener index, and species richness) in samples at different depths was quantified using the community ecology package ‘vegan 2.5-7’ attached to R software (Fig. 2e) [67].

### Measuring stable carbon isotopes

For a total of 19 lignite samples from XLT02 to XLT20, stable carbon isotope values were obtained by using the stable isotope ratio mass spectrometry method (Fig. 2e) [68]: (1) Sample preparation. Lignite samples of approximately 5 g were collected, cleaned to remove floating soil impurities on the surface, and then dried for 6 h in an oven at 60–70 °C. The fully dried sample was pounded to 0.250 mm (60 mesh) or less in a mortar and stored in a finger-like centrifuge tube for subsequent sampling. (2) Sample weighing. The carbon content of a single sample used for mass spectrometry was 20 mg. As lignite generally has a carbon content of approximately 60–77%, the single sample size is 26–34 mg. The sample to be weighed was wrapped in a tin boat to ensure that the package was correct. The sample was weighed the sample with a high-precision electronic scale. After weighing the sample in the tin bag, the bag was placed on a clean magnetic plate with bent tweezers. The tin boat containing the sample was folded into small squares and pressed tightly with tweezers. (3) Reaction. The small tin boat squares to be measured were successively placed in the mass spectrometer for the reaction. The instrument model was EA-DELTA plus XP with an accuracy of ≤ 0.2‰. The carbon isotopic ratios are reported in standard notation relative to the Vienna Pee Dee Belemnite (V-PDB) standard.

## Electronic supplementary material

Below is the link to the electronic supplementary material.

Supplementary Material 1

## Data Availability

All data generated or analysed during this study are included in this published article and its additional files.

## List of abbreviations

Ma: Million years ago
SEMTP: Southeastern margin of the Tibetan Plateau
XLT: Xiaolongtan
a.s.l.: Above sea level
YM: Yuanmou
CoA: Coexistence approach
MAT: Mean annual temperature
MAP: Mean annual precipitation
MWMT: Mean warmest monthly temperature
MCMT: Mean coldest monthly temperature
DT: The temperature difference between the coldest and warmest months
MMaP: Mean maximum monthly precipitation
MMiP: Mean minimum monthly precipitation.
