# Supplementary material for: Palaeobotanical evidence reveals the living conditions of Miocene Lufengpithecus in East Asia
Source: BMC Plant Biol. 2023 Mar 22;23:155. doi: 10.1186/s12870-023-04165-3 (PMC10031969; doi:10.1186/s12870-023-04165-3)
Supplement: Supplementary file 1 — Supplementary Material 1 [file 12870_2023_4165_MOESM1_ESM.docx]

**Supplementary Information for**

Palaeobotanical evidence reveals the living conditions of Miocene *Lufengpithecus* in East Asia

Li-Li Lu, Yi-Feng Yao, Guo-An Wang, Gan Xie, Kai-Qing Lu, Bin Sun, Jin-Feng Li, Angela A. Bruch, David K. Ferguson, Yi-Ming Cui, Qiang Wang, Xin-Ying Zhou, Feng Gao, Yu-Fei Wang

Email: zhouxinying@ivpp.ac.cn(X.-Y. Z.); yngaof@vip.sina.com (F. G.); wangyf@ibcas.ac.cn (Y.-F. W.)

**This supplementary file includes:**

Additional file 1: Additional information

Additional file 2: Fig. S1

Additional file 3: Fig. S2

Additional file 4: Fig. S3

Additional file 5: Fig. S4

Additional file 6: Fig. S5

Additional file 7: Fig. S6

Additional file 8: Table S1

Additional file 9: Table S2

Additional file 10: Table S3

Additional file 11: Table S4

Additional file 12: Table S5

Additional file 13: Table S6

Additional file 14: Table S7

Additional file 15: Table S8

Additional file 16: Table S9

Supplementary References

**Additional information**

**Palaeovegetation succession sequence in the Xiaolongtan section.**

Based on the changes in the relative abundance of dominant and/or characteristic groups in the pollen assemblages, the whole vegetation succession process can be divided into four phases corresponding to four zones in the pollen diagram (Fig. 2b and Additional file 6: Fig. S5).

**Phase 1** (corresponding to Zone I). The evergreen and deciduous broad-leaved mixed forest was largely composed of woody plants such as *Quercus, Juglans,* and *Carya,* which are common forest taxa in temperate to subtropical areas today. *Castanopsis*, as one of the main canopy species in today's hilly to subalpine evergreen broad-leaved forests, was also the dominant tree in this forest. There were many megathermic elements in the forest, such as Rutaceae, Aquifoliaceae, and Meliaceae. Some herbs, such as Lamiaceae, Brassicaceae, and Poaceae, grew in forest gaps. In addition, there were some shallow water plants, such as Potamogetonaceae and *Typha*, indicating the existence of water at the forest margin. For gymnosperms, there were a few warm and hygrophilous Taxodiaceae. A small amount of *Pinus* may have grown on distant mountains. Ferns, especially Polypodiaceae, thrived in the forest.

**Phase 2** (corresponding to Zone II). The vegetation still consisted of subtropical evergreen and deciduous broad-leaved mixed forest, and large stands of pure *Quercus* forest may have become established. They were interspersed with small amounts of *Juglans*, *Carya,* and *Castanopsis*. A small amount of hygrophilous *Alnus* appeared in this stage. The number of megathermic elements decreased, while the mega-mesothermic Euphorbiaceae increased. The abundance of herbaceous groups such as Poaceae and Lamiaceae increased significantly. In addition, the number of aquatic Potamogetonaceae diminished. *Pinus* forests expanded in the distant mountains. Ferns in the forest also decreased to a certain extent, but Polypodiaceae still flourished.

**Phase 3** (corresponding to Zone III). The forest was dominated by *Carya* with small numbers of *Quercus, Juglans, Castanea,* and *Ulmus*, as well as occasional *Castanopsis* and *Alnus*. The number of megathermic elements fell. The abundance of the megathermic element Aquifoliaceae increased slightly, and the number of mega-mesothermic elements Euphorbiaceae diminished slightly. The abundance of herbaceous taxa dominated by Poaceae and Lamiaceae decreased significantly. The number of aquatic Potamogetonaceae and *Typha* continued to fall. The number of *Pinus* on the distant mountain decreased slightly, and a small number of *Ephedra* grew on the edge of the forest. The abundance of ferns growing in the humid area increased, with Polypodiaceae still preeminent.

**Phase 4** (corresponding to Zone IV). In this phase, plant diversity declined and forest cover decreased. *Castanea* was the dominant tree, accompanied by Magnoliaceae and small amounts of *Carya, Quercus,* and *Juglans*; *Alnus* spread, while *Castanopsis* disappeared. The number of megathermic elements and mega-mesothermic elements continued to decrease, with the number of Aquifoliaceae falling slightly, while the number of Taxodiaceae increased. The abundance of herbaceous plants in the forest rose considerably, with Poaceae as the predominant group. The amount of *Pinus* declined slightly regionally, while the *Ephedra* at the forest margin increased somewhat. There was a decrease in ferns.

**Quantitative reconstruction of the palaeoclimate in the Xiaolongtan section.**

**Temperature parameters** (Fig. 2c-e, Additional file 10: Table S3). As delimiting taxa, *Carya* and *Ephedra* defined the range of MAT as 14.2-14.9 °C (median 14.6 °C) in each zone; today, the local MAT is 20.1 °C. As delimiting taxa, *Castanea* and Ranunculaceae determined the range of MWMT in Zone I and Zone III, and the overall range was 22.9-27.5 °C (median 25.2 °C). As delimiting taxa, *Castanea* and Asteraceae established the MWMT as 22.9 °C and that of Zone IV as 22.9-28.0 °C (median 25.5 °C), while the local MWMT is 24.8 °C today. As delimiting taxa, *Carya* and *Ephedra* demonstrated that the MCMT had a range of -0.3-5.9 °C (median 2.8 °C), with each zone consistent with the overall value; today, the local MCMT is 13.4 °C. As delimiting taxa, Solanaceae and *Castanopsis* established the DT range (in Zone I) as 14.0-26.0 °C (median 20.0 °C). In Zones II and III, *Ephedra* and *Castanopsis* fixed the DT ranges as 12.1-26.0 °C (median 19.1 °C). In Zone IV, the delimiting taxa *Ephedra* and *Carya* verified the DT ranges as 12.1-27.6 °C (median 19.9 °C); today, the local DT is 11.4 °C.

**Precipitation parameters** (Fig. 2c-e, Additional file 10: S3). *Castanopsis* and *Ephedra* established the MAP range as 614-1389 mm (median 1000 mm), with each zone being consistent with the overall values; today's local MAP is 770.6 mm. As delimiting taxa, *Carya* and *Ephedra* defined the range of MMaP as 142–206 mm (median 174 mm), with each zone being consistent with the overall values; today's local MMaP is 147 mm. As delimiting taxa, *Carya* and *Ephedra* determined the range of MMiP to be 7-24 mm (median 15 mm), with each zone being consistent with the overall values; today's local MMiP is 12.9 mm.

**Stable carbon isotopes in the Xiaolongtan section.**

Carbon isotope analysis is one of the important indices for the reconstruction of the palaeoenvironment [1]. The δ^13^C values of the samples show only slight fluctuations (Fig. 2e). In Zone I, the maximum value is -26.909 ‰ at XLT20, followed by a decline to the first low value of -28.015 ‰ at XLT18. After that, XLT17-XLT15 gradually increased to -27.088 ‰, but at the end of the zone, the trend was reversed with the lowest value of -28.332 ‰ being reached at XLT14. In Zone Ⅱ, the value rose to -27.149% at XLT13 and then decreased continuously. This downwards trend persisted in the upper part of Zone III (XLT10), where it reached the third minimum of -27.836%. Then, the δ^13^C increased gradually, followed by a decrease to -28.132 ‰, which was the second-lowest value in all samples. After this, there was a slight increase at XLT05 followed by a decline at XLT04 and XLT03. Zone IV is only represented by XLT02, in which δ^13^C greatly increased to the second-highest value of-27.021%. Since the range of variation in carbon isotope values throughout the section was not very large (1.4‰), we speculate that the climate during the *L*. *keiyuanensis* era was fairly stable with little fluctuation.

Most of the organic matter in sediments comes from terrestrial plant detritus. Due to carbon isotope fractionation during the degradation of organic matter, the carbon isotope composition of organic matter in sediments is more positive than that of vegetation isotopes [2-5]. The carbon isotopes of soil organic matter are generally more positive than those of plant isotopes by 2.3‰ (1.8‰-2.8‰) [6]. Given that the carbon isotopes of organic matter in our sediments lie between -26.91‰ and 28.33‰, with an average value of -27.60‰, we speculated that the carbon isotopes of surface vegetation at that time might have been between -29.21‰ and 30.63‰, with an average value of approximately -29.60‰. Because C_3_ and C_4_ plants have different carbon isotope fractionation mechanisms during photosynthesis [7, 8], there are significant differences in the carbon isotopic composition of C_3_ and C_4_ plants as a result. Thus, the global carbon isotope composition range of C_3_ plants is -20‰ − 35‰, with an average of -27‰; the range of C_4_ plants is -9‰−16‰, with a mean value of -13‰ [9]. Therefore, we conclude that all the terrestrial plants growing at that time must have been C_3_ plants.

Moreover, the carbon isotope composition of plants is also affected by other environmental factors, of which precipitation and temperature are the most important The influence of precipitation is usually greater than that of temperature. The carbon isotope composition of C_3_ plants has a significant negative correlation with annual precipitation [6, 10]. Based on our large sample survey of modern plants in China (unpublished data), C_3_ plant δ^13^C becomes increasingly negative with increasing precipitation and temperature from the arid area in Northwest China, the semiarid and semihumid area in North China, to the humid area on the southeast coast [11]. For example, the average value of C_3_ plant δ^13^C in the semiarid area of Northwest China (annual precipitation of 200-400 mm) is -26.1‰; the semihumid region of North China (400-600 mm annual precipitation) is 27.5 ‰; Guangyuan, Sichuan and Jianmen Pass (~ 900 mm annual precipitation) is 29.7 ‰; Zunyi, Guizhou and Zigong, Sichuan (~ 1100 mm annual precipitation) is -30.5 ‰; and Yibin, Sichuan and Xiuwen, Guizhou (~ 1300 mm annual precipitation) is -30.5 ‰. Based on the carbon isotope composition distribution of modern plants in China and a carbon isotope ratio of atmospheric CO_2_ before the industrial revolution of 1.3‰ [12, 13], we speculate that the annual precipitation of the local area in which *L. keiyuanensis* lived was at least 1000 mm.

**Palynological data in the Yuanmou section.**

The Miocene Yuanmou (YM) section (Additional file 7: Fig. S6, 25°54.792′N, 101°46.124′E, 1142 m a.s.l.) bearing the skull of *L. hudienensis* contains a highly diverse palynomorph assemblage of forty-three pollen and spore taxa (see more details in **Additional file 15: Table S8**). The assemblage is dominated by pollen of herbs/grasses (73.8%, mainly *Artemisia*) and woody plants (15.3%, mainly *Quercus* and *Juglans*) with abundant spores of ferns (7.45%), including Polypodiaceae and Athyriaceae during the existence of *L. hudienensis*.


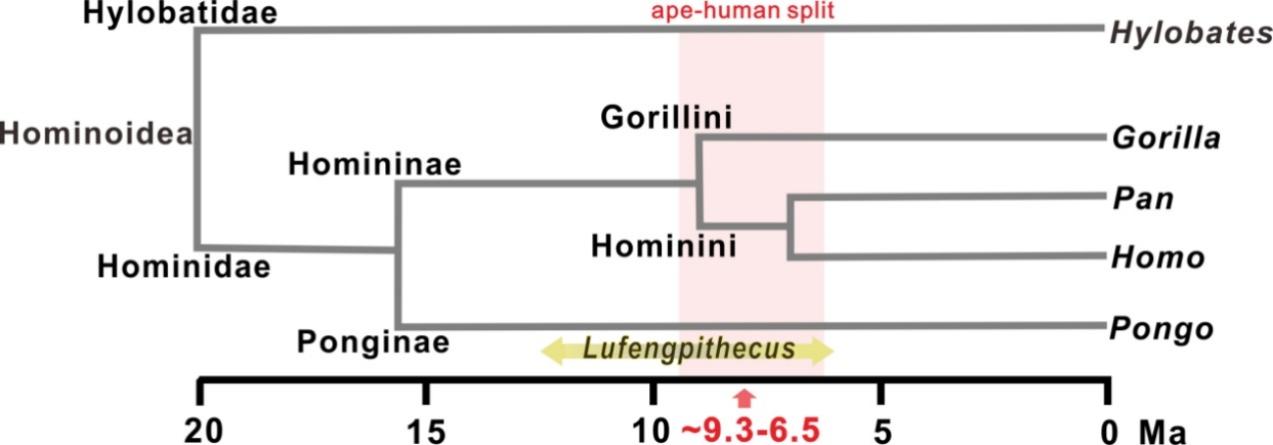


**Fig. S1. Phylogeny of Hominoidea (modified from TIME TREE,** [**http://www.timetree.org/**](http://www.timetree.org/)**).**


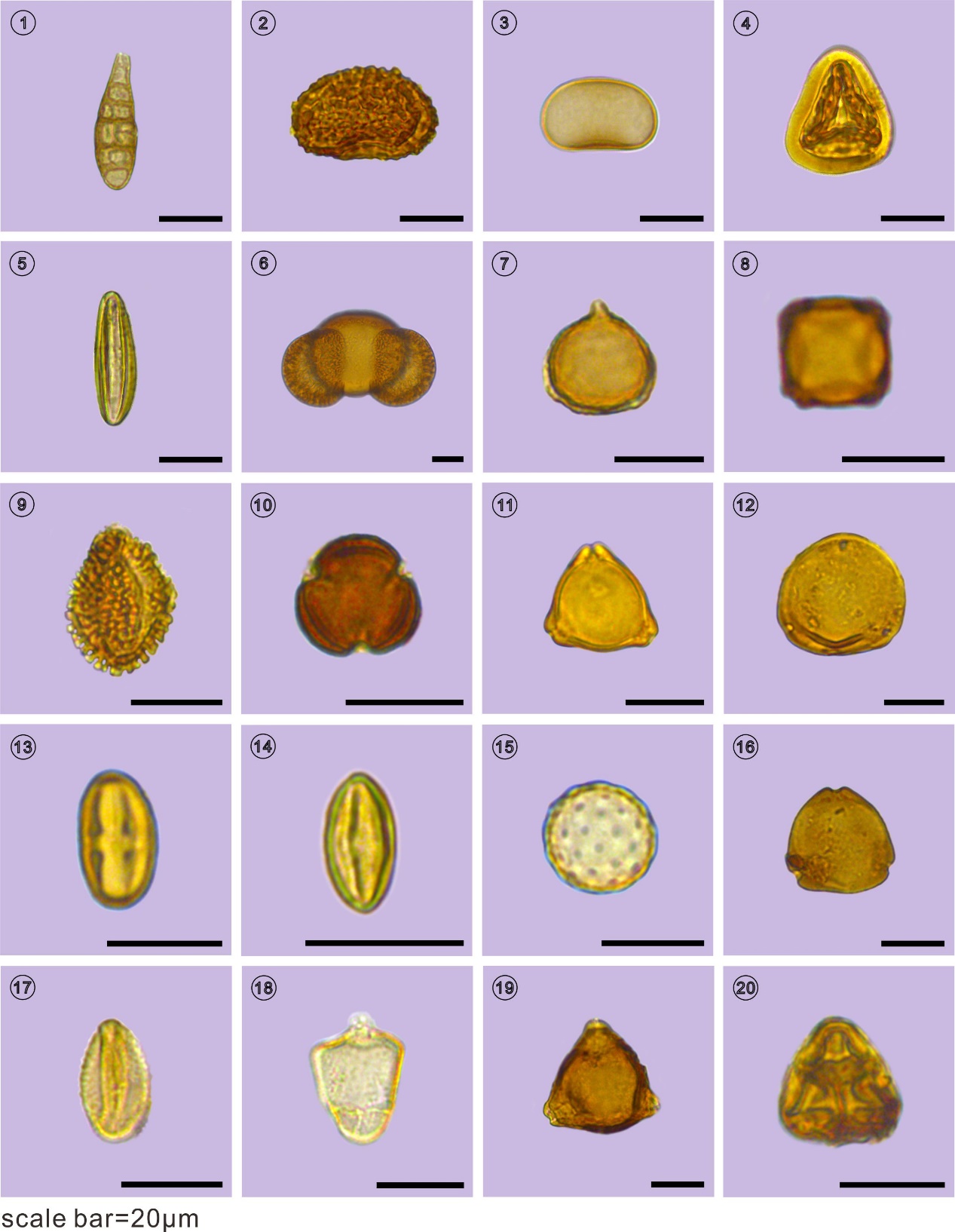


**Fig. S2. Major palynomorphs from the Xiaolongtan section. Scale bar = 20 μm.**

1. Fungi; 2. Athyriaceae; 3. Polypodiaceae; 4. Pteridaceae; 5. *Ephedra*; 6. *Pinus*; 7. Taxodiaceae; 8. *Alnus*; 9. Aquifoliaceae; 10. Artemisia; 11. *Betula*; 12. *Carya*; 13. *Castanea*; 14. *Castanopsis*; 15. Chenopodiaceae; 16. *Corylus*; 17. Brassicaceae; 18. Cyperaceae; 19. Elaeagnaceae; 20. Ericaceae.


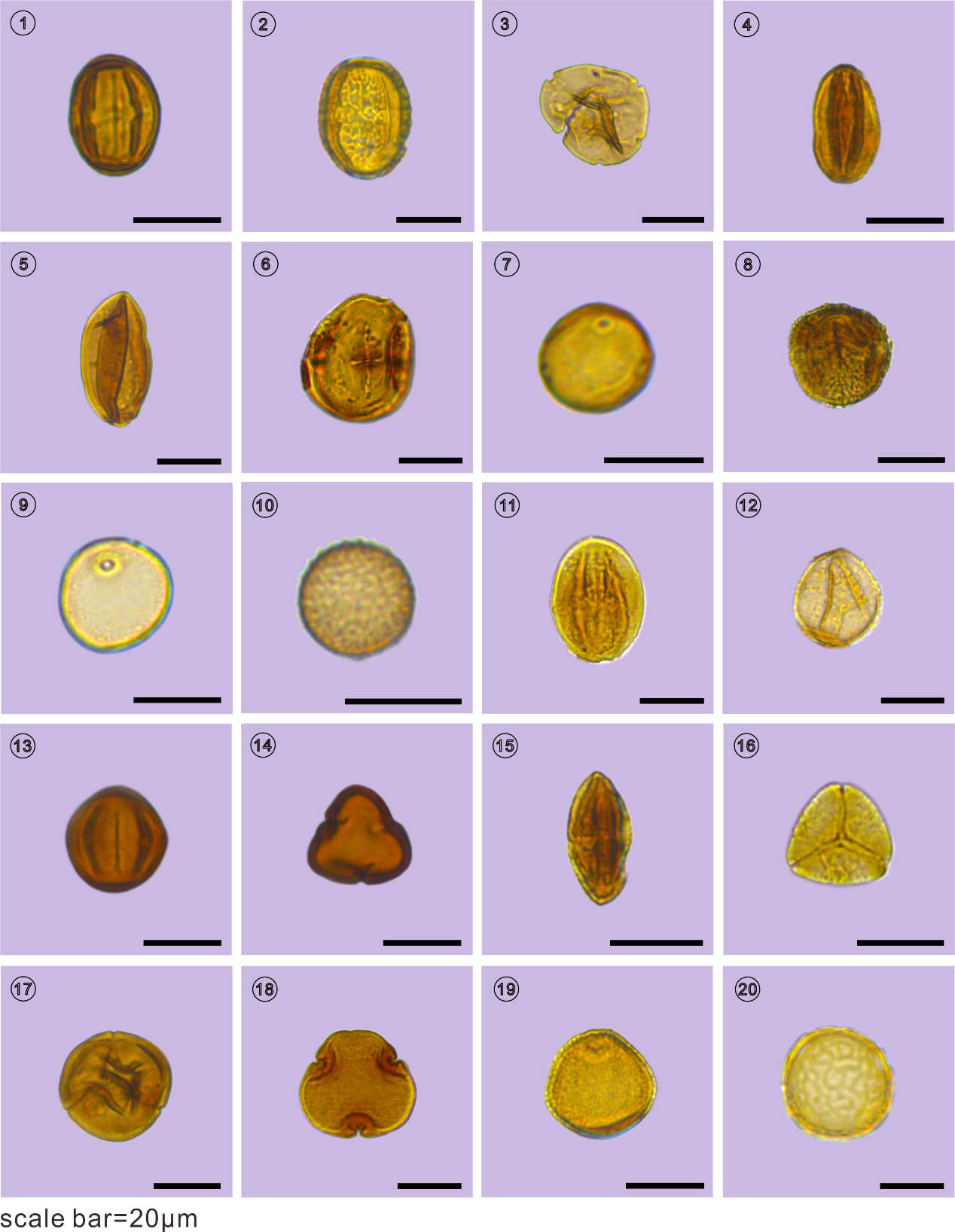


**Fig. S3. Major palynomorphs from the Xiaolongtan section. Scale bar = 20 μm.**

1. Euphorbiaceae; 2. Fabaceae; 3. *Juglans*; 4. Lamiaceae; 5. Magnoliaceae; 6. Meliaceae; 7. Moraceae; 8. Oleaceae; 9. Poaceae; 10. Potamogetonaceae; 11. *Quercus*; 12. Ranunculaceae; 13-14. Rosaceae; 15. Rutaceae; 16. Sapindaceae; 17. Solanaceae; 18. *Tilia*; 19. *Typha*; 20. *Ulmus.*


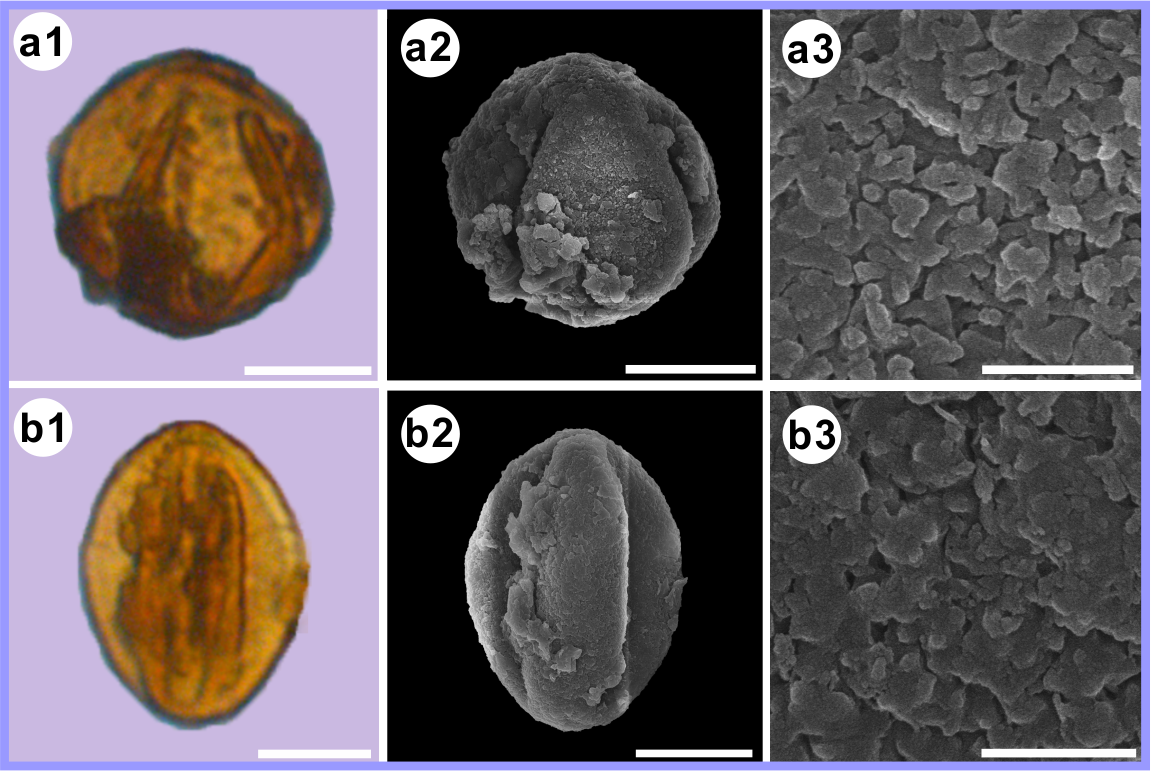


**Fig. S4. Photomicrographs of *Quercus* pollen grains.** Typical evergreen *Quercus* pollen grains with rod-like ornamentation under the light microscope (LM) (a1, b1) and under the scanning electron microscopic (SEM) (a2, a3, b2, b3). These identifications were based on ref.14.

Scale bar in LM and SEM overview 10 μm, in SEM close-up 1 μm.


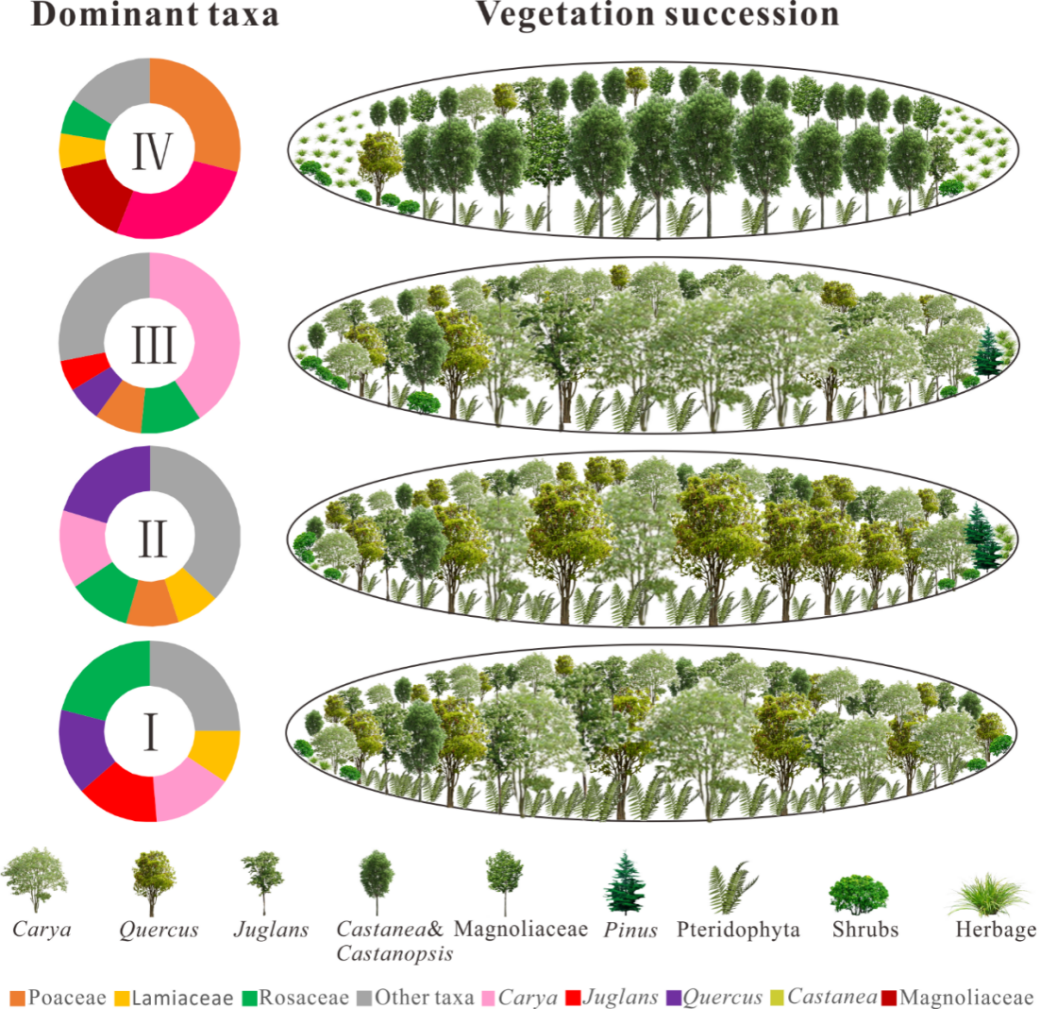


**Fig. S5. Vegetation succession from Zone I to Zone IV in the section from the Xiaolongtan Basin.**


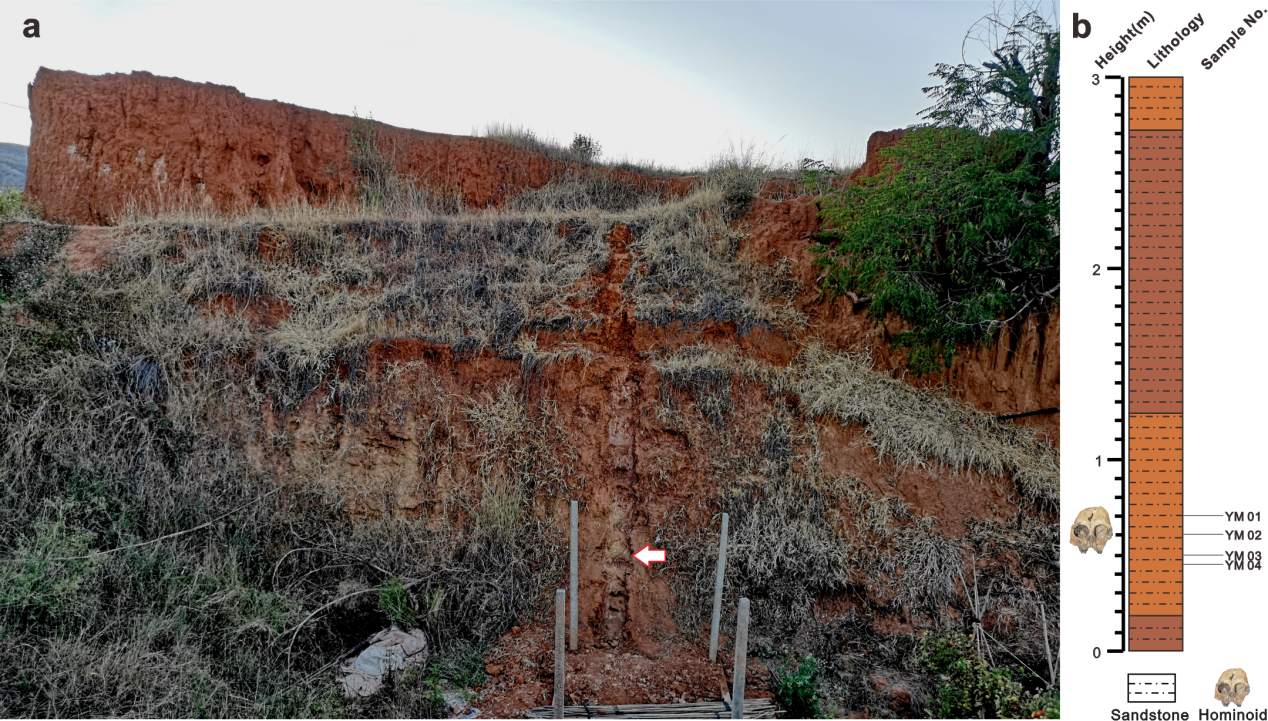


**Fig. S6. The sampling section and pollen sampling sequence in Yuanmou.** a. Photos of sampling locality bearing hominoid skull (indicated by the arrow) in Yuanmou. b. The measured stratigraphic sequence and pollen sampling sequence of the Yuanmou section.

**Table S1. Relative abundance of palynomorphs recovered from the Xiaolongtan section.**

| **Palynomorph relative abundance (%)** | | | |
| --- | --- | --- | --- |
| **Fungi** | **1.13** | Aquifoliaceae | 1.52 |
| **Algae** | **0.16** | Euphorbiaceae | 1.11 |
| **Moss** | **1.64** | *Ulmus* | 1.02 |
| **Pteridophytes** | **32.89** | Brassicaceae | 0.80 |
| Polypodiaceae | 27.13 | Rutaceae | 0.72 |
| Athyriaceae | 4.62 | Potamogetonaceae | 0.65 |
| Pteridophytes (others) | 0.54 | *Alnus* | 0.54 |
| Dennstaedtiaceae | 0.30 | *Corylus* | 0.51 |
| Selaginellaceae | 0.18 | Fabaceae | 0.50 |
| Pteridaceae | 0.11 | *Betula* | 0.47 |
| Davalliaceae | 0.02 | Meliaceae | 0.39 |
| **Gymnosperms** | **2.00** | *Typha* | 0.39 |
| *Pinus* | 1.86 | Ranunculaceae | 0.33 |
| *Ephedra* | 0.09 | Ericaceae | 0.30 |
| Taxodiaceae | 0.05 | Chenopodiaceae | 0.27 |
| **Angiosperms** | **62.18** | *Artemisia* | 0.23 |
| *Carya* | 14.19 | Araliaceae | 0.15 |
| Rosaceae | 9.04 | Asteraceae (excl. *Artemisia*) | 0.12 |
| *Quercus* | 7.28 | *Tilia* | 0.12 |
| Poaceae | 5.37 | Moraceae | 0.09 |
| *Juglans* | 5.24 | Oleaceae | 0.09 |
| Lamiaceae | 4.37 | Elaeagnaceae | 0.06 |
| *Castanea* | 2.75 | Solanaceae | 0.06 |
| *Castanopsis* | 1.94 | Sapindaceae | 0.05 |
| Magnoliaceae | 1.53 | Cyperaceae | 0.02 |

**Table S2. List of the palynomorphs grouped by ecological requirements and their relative abundances in the four pollen zones of the Xiaolongtan section (the assignment of taxa to ecological groups follows ref.15).**

| **Zones** | **I (%)** | **II (%)** | **III (%)** | **IV (%)** |
| --- | --- | --- | --- | --- |
| **Megathermic elements** |  |  |  |  |
| Aquifoliaceae | 0.88 | 0.50 | 2.89 | 1.45 |
| Meliaceae | 0.38 | 0.66 | 0.40 | - |
| Rutaceae | 1.04 | 0.66 | 0.67 | - |
| Sapindaceae | 0.08 | 0.08 | - | - |
| Solanaceae | 0.08 | - | - | 0.32 |
| **Mega-mesothermic elements** |  |  |  |  |
| Araliaceae | 0.08 | 0.66 | - | - |
| *Castanopsis* | 2.92 | 2.64 | 1.20 | - |
| Euphorbiaceae | 1.00 | 4.04 | 0.04 | - |
| Taxodiaceae | 0.08 | - | - | 0.16 |
| **Mesothermic elements** |  |  |  |  |
| *Alnus* | - | 1.90 | 0.22 | 1.29 |
| *Betula* | 0.08 | 0.25 | 1.16 | - |
| *Carya* | 9.09 | 8.83 | 27.32 | 0.80 |
| *Castanea* | 0.79 | 0.50 | 1.20 | 21.06 |
| *Corylus* | 0.38 | 0.83 | 0.67 | - |
| Elaeagnaceae | 0.17 | - | - | - |
| *Juglans* | 9.30 | 2.97 | 3.69 | 1.13 |
| Magnoliaceae | - | - | 1.16 | 12.22 |
| Moraceae | - | 0.41 | 0.04 | - |
| Oleaceae | 0.21 | - | - | 0.16 |
| *Quercus* | 9.63 | 12.79 | 4.26 | 0.48 |
| *Tilia* | 0.17 | 0.17 | 0.09 | - |
| *Ulmus* | 0.79 | 0.41 | 1.87 | 0.32 |
| **Meso-microthermic elements** |  |  |  |  |
| *Pinus* | 0.58 | 3.47 | 2.84 | 0.64 |
| **Nonsignificant elements** |  |  |  |  |
| Rosaceae | 13.34 | 7.10 | 7.33 | 4.98 |
| **Herbs and Shrubs** |  |  |  |  |
| *Artemisia* | 0.08 | - | 0.04 | 1.93 |
| Asteraceae (excl. *Artemisia*) | 0.17 | 0.08 | 0.13 | - |
| Brassicaceae | 1.63 | 0.17 | 0.13 | 1.45 |
| Chenopodiaceae | 0.13 | 0.08 | 0.13 | 1.77 |
| *Ephedra* | 0.08 | 0.17 | 0.04 | 0.16 |
| Ericaceae | - | 1.49 | 0.04 | 0.16 |
| Fabaceae | 0.58 | 0.50 | 0.58 | - |
| Lamiaceae | 5.92 | 4.87 | 2.62 | 4.98 |
| Poaceae | 0.67 | 5.86 | 5.69 | 22.99 |
| Ranunculaceae | 0.58 | - | 0.36 | - |

Continued Table S2

| **Zones** | **I (%)** | **II (%)** | **III (%)** | **IV (%)** |
| --- | --- | --- | --- | --- |
| **Aquatic macrophytes** |  |  |  |  |
| Cyperaceae | - | - | 0.04 | - |
| Potamogetonaceae | 1.46 | 0.25 | 0.22 | - |
| *Typha* | 0.58 | 0.41 | 0.22 | 0.32 |
| **Pteridophytes** |  |  |  |  |
| Athyriaceae | 7.92 | 5.20 | 2.18 | 0.96 |
| Davalliaceae | 0.04 | - | - | - |
| Dennstaedtiaceae | 0.08 | - | 0.53 | 0.96 |
| Polypodiaceae | 27.97 | 32.26 | 28.83 | 15.43 |
| Pteridaceae | 0.13 | 0.25 | 0.04 | - |
| Selaginellaceae | - | 0.41 | 0.31 | - |
| Ferns (other) | 1.17 | 0.08 | 0.31 | - |
| **Other elements** |  |  |  |  |
| Moss | 0.13 | 1.82 | 2.89 | 3.05 |
| Algae | 0.17 | 0.33 | 0.13 | - |
| Fungi | 0.21 | 0.17 | 2.22 | 2.89 |
| unknown | 1.33 | 0.83 | 0.71 | 0.16 |

-: no pollen grains

**Table S3. List of the differences and similarities based on palynological assemblages of Zones I and III, the possible horizons bearing *L. keiyuanensis***

| **Characteristics of palynological assemblages** | | **Zone I** | **Zone III** |
| --- | --- | --- | --- |
| Differences | Dominant components in trees | *Quercus* (9.6%), *Juglans* (9.1%) and *Carya* (8.9%) | *Carya* (26.3%), *Quercus* (3.6%) and *Juglans* (3.1%) |
|  | Dominant components in herbs | Lamiaceae (5.8%) | Poaceae (4.8%) and Lamiaceae (2.3%) |
|  |  | Polypodiaceae (27.4%) | Polypodiaceae (30.5%) |
|  | Megathermic elements | Aquifoliaceae (0.88%), Meliaceae (0.38%), Rutaceae (1.04%), Sapindaceae (0.08%), and Solanaceae (0.08%) | Aquifoliaceae (2.89), Meliaceae (0.40), and Rutaceae (0.67) |
|  | Mega-mesothermic elements | Araliaceae (0.08%), *Castanopsis* (2.92%), Euphorbiaceae (1.00%), and Taxodiaceae (0.08%) | *Castanopsis* (1.20%) and Euphorbiaceae (0.04%) |
| Similarities | | Both of them indicate evergreen and deciduous broad-leaved mixed forests. | |

**Table S4. Palaeoclimatic parameters of the whole pollen assemblage and each of the four pollen zones of the Xiaolongtan section by using the CoA (modern data from National Meteorological Information Center,** [**http://data.cma.cn/**](http://data.cma.cn/)**).**

|  | **Total** | **Zone I** | **Zone II** | **Zone III** | **Zone IV** | **Modern** |
| --- | --- | --- | --- | --- | --- | --- |
| MAT (°C) | 14.6 (14.2-14.9) | 14.6 (14.2-14.9) | 14.6 (14.2-14.9) | 14.6 (14.2-14.9) | 14.6 (14.2-14.9) | 20.1 |
| Delimiting taxa | *Carya*, *Ephedra* | *Carya*, *Ephedra* | *Carya*, *Ephedra* | *Carya*, *Ephedra* | *Carya*, *Ephedra* | - |
| MWMT (°C) | 25.2 (22.9-27.5) | 25.2 (22.9-27.5) | 25.5 (22.9-28.0) | 25.2 (22.9-27.5) | 25.5 (22.9-28.0) | 24.8 |
| Delimiting taxa | *Castanea*, Ranunculaceae | *Castanea*, Ranunculaceae | *Castanea*, Asteraceae | *Castanea*, Ranunculaceae | *Castanea*, Asteraceae | - |
| MCMT (°C) | 2.8 (-0.3-5.9) | 2.8 (-0.3-5.9) | 2.8 (-0.3-5.9) | 2.8 (-0.3-5.9) | 2.8 (-0.3-5.9) | 13.4 |
| Delimiting taxa | *Carya*, *Ephedra* | *Carya*, *Ephedra* | *Carya*, *Ephedra* | *Carya*, *Ephedra* | *Carya*, *Ephedra* | - |
| DT (°C) | 20.0 (14.0-26.0) | 20.0 (14.0-26.0) | 19.1 (12.1-26.0) | 19.1 (12.1-26.0) | 19.9（12.1-27.6） | 11.4 |
| Delimiting taxa | Solanaceae, *Castanopsis* | Solanaceae, *Castanopsis* | *Ephedra*, *Castanopsis* | *Ephedra*, *Castanopsis* | *Ephedra*, *Carya* | - |
| MAP (mm) | 1002 (614-1389) | 1002 (614-1389) | 1002 (614-1389) | 1002 (614-1389) | 995 (601-1389) | 770.6 |
| Delimiting taxa | *Castanopsis*, *Ephedra* | *Castanopsis*, *Ephedra* | *Castanopsis*, *Ephedra* | *Castanopsis*, *Ephedra* | *Carya*, *Ephedra* | - |
| MMaP (mm) | 174 (142-206) | 174 (142-206) | 174 (142-206) | 174 (142-206) | 174 (142-206) | 147 |
| Delimiting taxa | *Carya*, *Ephedra* | *Carya*, *Ephedra* | *Carya*, *Ephedra* | *Carya*, *Ephedra* | *Carya*, *Ephedra* | - |
| MMiP (mm) | 15 (7-24) | 15 (7-24) | 15 (7-24) | 15 (7-24) | 15 (7-24) | 12.9 |
| Delimiting taxa | *Carya*, *Ephedra* | *Carya*, *Ephedra* | *Carya*, *Ephedra* | *Carya*, *Ephedra* | *Carya*, *Ephedra* | - |

**Table S5. The detailed differences between this study and Xia et al. [16]**

|  | **This study** | **Xia et al., 2009 [16]** |
| --- | --- | --- |
| **Location** | Xiaolongtan Basin, 23°48.351′ N, 103°10.410′ E, 1105 m a.s.l. | Xiaolongtan Basin, 23°48.75′ N, 103°11.867′ E, 1050 m a.s.l. |
| **Layers** | Covering the excavation position of the Lufengpithecus keiyuanensis fossils which may be located at the upper or lower part of the lignite of Xiaolongtan Formation | Top layer of lignite of Xiaolongtan Formation |
| **Ages** | 12.5-11.6 Ma (Middle Miocene) | Late Miocene (cited from ref.17) |
| **Data resources** | Fossil pollen | Plant megafossils |
| **Number of fossil taxa** | 37 | 42 |
| **Qualitative palaeclimate** | Warm and humid subtropical climate | Humid subtropical climate |
| **Method of palaeoclimate calculation** | CoA | CoA, LMA, and CLAMP |
| **Database of NLRs used in CoA** | Palaeoflora database in East Asia | Palaeoflora database in Europe |
| **Quantitative palaeclimatic parameters** | MAT: 14.2-14.9 °C | MAT: 16.7-19.2 °C (CoA);  22.3 ± 2.05 °C (LMA);  18.1 ± 1.2 °C (CLAMP) |
|  | MAP: 614-1389 mm | MAP: 1215–1639 mm (CoA); 1964.8± 335.9 mm (CLAMP) |

**Table S6. Dominant fossil plant taxa and their fruit types at four *Lufengpithecus* sites in Yunnan.**

|  | **Xiaolongtan** | **Yuanmou** | **Lufeng** | **Zhaotong** |
| --- | --- | --- | --- | --- |
| **Dominant taxa** | **Pollen:**  *Carya, Quercus, Juglans, Castanea*;  **Mega-fossil:**  Fabaceae, Fagaceae, Lauraceae | **Pollen**: *Pterocarya, Ulmus, Quercus, Castanea, Juglans* | **Pollen**:  *Castanopsis, Myrica, Alnus, Quercus, Carpinus, Corylus* | **Pollen:**  *Quercus, Castanea, Castanopsis, Alnus*;  **Mega-fossil:**  *Carya, Corylus, Euryale, Trapa* |
| **Fruits types** | drupe, nut, pome, cone, pod | drupe, nut | drupe, nut | drupe, nut |
| **Age (Ma)** | 12.5-11.6 | ~9.0-7.1 | 6.9-6.2 | ~6.2 |
| **References** | 16, this study | 18 | 19 | 20, 21 |

**Table S7. Summary of Miocene hominoid sites around the Tibetan Plateau.**

| **Species** | **Fossil Site** | **Age (Ma)** | **Palaeovegetation** | **Palaeoclimatic parameters in hominoid living periods** | **Refs.** |
| --- | --- | --- | --- | --- | --- |
| *Sivapithecus* sp. | Siwalik | 12.7-6.8 | Evergreen broad-leaved forest dominated in the Middle Miocene, mixed evergreen deciduous broad-leaved forest dominated in the Late Miocene | ~13-11 Ma: MAT=21.1-25.4 °C, MAP=1748-2869 mm;  9.5-6.8 Ma: MAT=26-27 °C, MAP=2592-3151 mm | 22-24 |
| *Lufengpithecus keiyuanensis* | Xiaolongtan | 12.5-11.6 | Tropical - subtropical subhumid evergreen broad-leaved forest | MAT=14.2-14.9 °C; MAP=614-1389 mm | 16, 25, 26, this study |
| *Lufengpithecus hudienensis* | Yuanmou | ~9.0-7.1 | Subtropical mountain forest with dense forests and local transitional shrubs | MAT=14.2-14.9 °C; MAP=614-1389 mm | 18, 27, 28, this study |
| *Lufengpithecus lufengensis* | Lufeng | 6.9-6.2 | Tropical-subtropical monsoon evergreen and deciduous broad-leaved mixed forest with *Myrica* and *Alnus* as dominant taxa | MAT=14.2-14.9 °C; MAP=996-1389 mm | 19, 29, 30, this study |
| *Lufengpithecus* cf. *lufengensis* | Zhaotong | ~6.2 | Tropical-subtropical evergreen broad-leaved forest dominated by evergreen *Quercus* | MAT=11.3-17.6 °C; MAP=1042-1547 mm | 20, 21 |
| *Lufengpithecus* sp. | Baoshan | ~6.0 | - | *-* | 31 |
| *Khoratpithecus chiangmuanensis* | Chiang Muan | 13.5-10 | Evergreen and deciduous broad-leaved mixed forest dominated by *Syzygium* | - | 32-34 |
| *Khoratpithecus ayeyarwadyensis* | Myanmar | 10.4-8.8 | Evergreen forest | - | 35 |
| *Khoratpithecus piriyai* | Khorat | 9.0-7.0 | Gallery forest dominated by *Syzygium* and including other tropical taxa such as *Alchornea*, *Nauclea*, and the Caesalpiniaceae, Combretaceae, and Malpighiaceae | - | 33, 34 |

**Table S8. Reconstructed palaeoclimatic parameters of four *Lufengpithecus* sites on the southeastern margin of the Tibetan Plateau using the CoA.**

|  | **Xiaolongtan** | **Yuanmou** | **Lufeng** | **Zhaotong** |
| --- | --- | --- | --- | --- |
| **Age (Ma)** | 12.5-11.6 | ~9.0-7.1 | 6.9-6.2 | ~6.2 |
| **MAT (°C)** | 14.2-14.9 | 14.2-14.9 | 14.2-14.9 | 11.3-17.6 |
| **MWMT (°C)** | 22.9-27.5 | 22.9-27.5 | 23.8-25.4 | 21.9-27.5 |
| **MCMT (°C)** | -0.3-5.9 | -0.3-5.9 | 2.5-5.9 | 0.1-7.1 |
| **MAP (mm)** | 614-1389 | 614-1389 | 996-1389 | 1042-1547 |
| **MMaP (mm)** | 142-206 | 142-206 | 179-245 | 141-212 |
| **MMiP (mm)** | 7-24 | 7-24 | 11-20 | 8-55 |
| **References** | this study | this study | 19, this study | 21 |

**Table S9. Relative abundance of palynomorphs recovered from the Yuanmou section.**

| **Relative abundance (%)** | | | |
| --- | --- | --- | --- |
| **Algae** | **0.23** | *Artemisia* | 65.19 |
| **Fungi** | **1.28** | *Quercus* | 3.73 |
| **Moss** | **2.21** | Asteraceae (excl. *Artemisia*) | 2.68 |
| **Pteridophytes** | **7.45** | Poaceae | 2.68 |
| Polypodiaceae | 1.63 | Chenopodiaceae | 1.75 |
| Athyriaceae | 0.58 | Euphorbiaceae | 1.05 |
| Pteridaceae | 2.44 | *Juglans* | 0.93 |
| Dennstaedtiaceae | 2.79 | *Corylus* | 0.81 |
| **Gymnosperms** | **5.47** | *Castanea* | 0.70 |
| Cupressaceae s.s. | 1.98 | *Castanopsis* | 0.70 |
| *Pinus* | 1.51 | Fabaceae | 0.58 |
| Taxodiaceae | 0.58 | Lamiaceae | 0.58 |
| *Picea* | 0.58 | *Ulmus* | 0.58 |
| *Cedrus* | 0.35 | Polygonaceae | 0.47 |
| *Ephedra* | 0.23 | Moraceae | 0.35 |
| *Abies* | 0.12 | Rosaceae | 0.35 |
| *Tsuga* | 0.12 | *Carya* | 0.12 |
| **Angiosperms** | **86.03** | Elaeagnaceae | 0.12 |

**References**

1. Cerling TE, Wang Y, Quade J: **Expansion of C_4_ Ecosystems as an Indicator of Global Ecological Change in the Late Miocene**. *Nature* 1993, **361**(6410):344-345.

2. Ågren GI, Bosatta E, Balesdent J: **Isotope Discrimination during Decomposition of Organic Matter: A Theoretical Analysis**. *Soil Sci Soc Am J* 1996, **60**(4):1121-1126.

3. Feng XH: **A theoretical analysis of carbon isotope evolution of decomposing plant litters and soil organic matter**. *Global Biogeochem Cycles* 2002, **16**(4):66-61-66-11.

4. Poage MA, Feng XH: **A theoretical analysis of steady state δ 13 C profiles of soil organic matter**. *Global Biogeochem Cycles* 2004, **18**(2):1-13.

5. Wang GA, Jia YF, Li W: **Effects of environmental and biotic factors on carbon isotopic fractionation during decomposition of soil organic matter**. *Sci Rep* 2015, **5**:11043.

6. Wang G, Feng X, Han J, Zhou L, Tan W, Su F: **Paleovegetation reconstruction using δ13C of Soil Organic Matter**. *Biogeosciences* 2008, **5**(5):1325-1337.

7. Farquhar GD, O'Leary MH, Berry JA: **On the relationship between carbon isotope discrimination and the intercellular carbon dioxide concentration in leaves**. *Functional Plant Biology* 1982, **9**(2):121-137.

8. Farquhar GD: **On the nature of carbon isotope discrimination in C_4_ species**. *Aust J Plant Physiol* 1983, **10**(2):205-226.

9. Deines P: **The isotopic composition of reduced organic carbon**. In: *The Terrestrial Environment, A.* Edited by Fritz P, Fontes JC. Amsterdam: Elsevier; 1980: 329-406.

10. Diefendorf AF, Mueller KE, Wing SL, Koch PL, Freeman KH: **Global patterns in leaf ^13^C discrimination and implications for studies of past and future climate**. *Proc Natl Acad Sci U S A* 2010, **107**(13):5738-5743.

11. Wang GA, Han JM, Liu DS: **The carbon isotope composition of C3 herbaceous plants in loess area of northern China**. *Science in China Series D-Earth Sciences* 2003, **46**(10):1069-1076.

12. Leuenberger M, Siegenthaler U, Langway CC: **Carbon isotope composition of atmospheric CO2 during the Last Ice-Age from an Antarctic ice core**. *Nature* 1992, **357**(6378):488-490.

13. Marino BD, Mcelroy MB, Salawitch RJ, Spaulding WG: **Glacial-to-interglacial variations in the carbon isotopic composition of atmospheric CO2**. *Nature* 1992, **357**(6378):461-466.

14. Zhang X, Huang KY, Zheng Z, Zhang YZ, Wan QC, Tian LP: **Pollen morphology of *Quercus* sect. *Ilex* and its relevance for fossil pollen identification in southwest China**. *Grana* 2018, **57**(6):401-414.

15. Jiménez-Moreno G: **Progressive substitution of a subtropical forest for a temperate one during the middle Miocene climate cooling in Central Europe according to palynological data from cores Tengelic-2 and Hidas-53 (Pannonian Basin, Hungary)**. *Review of Palaeobotany and Palynology* 2006, **142**(1):1-14.

16. Xia K, Su T, Liu YS, Xing YW, Jacques FMB, Zhou ZK: **Quantitative climate reconstructions of the late Miocene Xiaolongtan megaflora from Yunnan, Southwest China**. *Palaeogeogr Palaeoclimatol Palaeoecol* 2009, **276**(1-4):80-86.

17. Wang WM: **A palynological survey of Neogene strata in Xiaolongtan basin, Yunnan Province of South China**. *Acta Bot Sin* 1996, 38(9):743-748.

18. Qian F, Ling XH: **The living environment and age of Yuanmou hominoid**. *J Chengdu Uni Technol (Sci Technol Ed)* 1998(2):311-318.

19. Sun XJ, Wu YS: **Deducing the natural environment during the life period of Lufeng *Ramapithecus* based on palynology**. *Journal of Vertebrate Paleontology* 1980, **18**(3):247-255.

20. Chang L, Guo ZT, Deng CL, Wu HB, Ji XP, Yan Z, Zhang CX, Ge JY, Wu BL, Sun L *et al*: **Pollen evidence of the palaeoenvironments of *Lufengpithecus lufengensis* in the Zhaotong Basin, southeastern margin of the Tibetan Plateau**. *Palaeogeogr Palaeoclimatol Palaeoecol* 2015, **435**(435):95-104.

21. Huang YJ, Ji XP, Su T, Deng CL, Ferguson DK, Yu TS, Yang X, Sun H, Zhou ZK: **Habitat, climate and potential plant food resources for the late Miocene Shuitangba hominoid in Southwest China: Insights from carpological remains**. *Palaeogeogr Palaeoclimatol Palaeoecol* 2017, **470**(1):63-71.

22. Begun D, Ward C, Rose M: **Function, phylogeny and fossils: Miocene hominoid origins and adaptations**; 1997.

23. Hoorn C, Ohja T, Quade J: **Palynological evidence for vegetation development and climatic change in the Sub-Himalayan Zone (Neogene, Central Nepal)**. *Palaeogeogr Palaeoclimatol Palaeoecol* 2000, **163**(3):133-161.

24. Srivastava G, Paudayal KN, Utescher T, Mehrotra RC: **Miocene vegetation shift and climate change: Evidence from the Siwalik of Nepal**. *Glob Planet Change* 2018, **161**:108-120.

25. Dong W: **Upper Cenozoic strata and paleoenvironment of Xiaolongtan Basin, Kaiyuan, Yunnan**. In: *Annual Conference of Chinese Society of Vertebrate Paleontology: 2001*.

26. Li SH, Deng CL, Dong W, Sun L, Liu SZ, Qin HF, Yin JY, Ji XP, Zhu RX: **Magnetostratigraphy of the Xiaolongtan Formation bearing *Lufengpithecus keiyuanensis* in Yunnan, Southwestern China: Constraint on the initiation time of the southern segment of the Xianshuihe-Xiaojiang fault**. *Tectonophysics* 2015, **655**(1):213-226.

27. Zhu RX, Liu QS, Yao HT, Guo ZT, Deng CL, Pan YX, Lu LQ, Chang ZG, Gao F: **Magnetostratigraphic dating of hominoid-bearing sediments at Zhupeng, Yuanmou Basin, southwestern China**. *Earth Planet Sci Lett* 2005, **236**(3):559-568.

28. Dong W, Qi GQ: **Hominoid-producing localities and biostratigraphy in Yunnan**. In: *Fossil Mammals of Asia: Neogene Biostratigraphy and Chronology.* Edited by Wang X, Flynn LJ, Fortelius M: Colombia University Press; 2013: 293-313.

29. Chen WY, Lin YF, Yu QL: **The paleoclimate during the period of *Ramapithecus* in Lufeng County, Yunnan Province**. *Acta Anthropol Sin* 1986, **5**(1):79-88.

30. Kelley J, Gao F: **Juvenile hominoid cranium from the late Miocene of southern China and hominoid diversity in Asia**. *Proc Natl Acad Sci U S A* 2012, **109**(18):6882-6885.

31. Li SH, Ji XP, Harrison T, Deng CL, Wang SQ, Wang LR, Zhu RX: **Uplift of the Hengduan Mountains on the southeastern margin of the Tibetan Plateau in the late Miocene and its paleoenvironmental impact on hominoid diversity**. *Palaeogeogr Palaeoclimatol Palaeoecol* 2020, **553**:14.

32. Chaimanee Y, Jolly D, Benammi M, Tafforeau P, Duzer D, Moussa I, Jaeger JJ: **A Middle Miocene hominoid from Thailand and orangutan origins**. *Nature* 2003, **422**(6927):61-65.

33. Chaimanee Y, Suteethorn V, Jintasakul P, Vidthayanon C, Marandat B, Jaeger JJ: **A new orang-utan relative from the Late Miocene of Thailand**. *Nature* 2004, **427**(6973):439-441.

34. Sepulchre P, Jolly D, Ducrocq S, Chaimanee Y, Jaeger JJ, Raillard A: **Mid-Tertiary paleoenvironments in Thailand: Pollen evidence**. *Clim Past* 2010, **6**(4):461-473.

35. Jaeger JJ, Soe AN, Chavasseau O, Coster P, Emonet EG, Guy F, Lebrun R, Maung A, Aung Khyaw A, Shwe H *et al*: **First hominoid from the Late Miocene of the Irrawaddy Formation (Myanmar)**. *PLoS ONE* 2011, **6**(4):e17065.
